# Genomic analysis of the native European *Solanum* species, *S. dulcamara*

**DOI:** 10.1186/1471-2164-14-356

**Published:** 2013-05-28

**Authors:** Nunzio D’Agostino, Tomek Golas, Henri van de Geest, Aureliano Bombarely, Thikra Dawood, Jan Zethof, Nicky Driedonks, Erik Wijnker, Joachim Bargsten, Jan-Peter Nap, Celestina Mariani, Ivo Rieu

**Affiliations:** 1 IWWR, Department of Molecular Plant Physiology, Radboud University Nijmegen, Heyendaalseweg 135, Nijmegen, 6525 AJ, The Netherlands; 2 Consiglio per la ricerca e la sperimentazione in agricoltura, Centro di ricerca per l’orticoltura, via Cavalleggeri 25, Pontecagnano, SA, 84098, Italy; 3 Applied Bioinformatics, Bioscience, Plant Research International, Wageningen University & Research Centre, PO Box 619, Wageningen, 6700 AP, The Netherlands; 4 Boyce Thompson Institute for Plant Research, Tower Road, Ithaca, New York, 14853-1801, USA; 5 Laboratory of Genetics, Wageningen University, Droevendaalsesteeg 1, Wageningen, 6708 PB, the Netherlands; 6 Centre for BioSystems Genomics 2012 (CBSG2012), PO Box 98, Wageningen, 6700 AB, The Netherlands

**Keywords:** *Solanum dulcamara*, Bittersweet, *de novo* transcriptome assembly, Genetic map, Comparative genomics

## Abstract

**Background:**

*Solanum dulcamara* (bittersweet, climbing nightshade) is one of the few species of the Solanaceae family native to Europe. As a common weed it is adapted to a wide range of ecological niches and it has long been recognized as one of the alternative hosts for pathogens and pests responsible for many important diseases in potato, such as *Phytophthora*. At the same time, it may represent an alternative source of resistance genes against these diseases. Despite its unique ecology and potential as a genetic resource, genomic research tools are lacking for *S. dulcamara*. We have taken advantage of next-generation sequencing to speed up research on and use of this non-model species.

**Results:**

In this work, we present the first large-scale characterization of the *S. dulcamara* transcriptome. Through comparison of RNAseq reads from two different accessions, we were able to predict transcript-based SNP and SSR markers. Using the SNP markers in combination with genomic AFLP and CAPS markers, the first genome-wide genetic linkage map of bittersweet was generated. Based on gene orthology, the markers were anchored to the genome of related *Solanum* species (tomato, potato and eggplant), revealing both conserved and novel chromosomal rearrangements. This allowed a better estimation of the evolutionary moment of rearrangements in a number of cases and showed that chromosomal breakpoints are regularly re-used.

**Conclusion:**

Knowledge and tools developed as part of this study pave the way for future genomic research and exploitation of this wild *Solanum* species. The transcriptome assembly represents a resource for functional analysis of genes underlying interesting biological and agronomical traits and, in the absence of the full genome, provides a reference for RNAseq gene expression profiling aimed at understanding the unique biology of *S. dulcamara*. Cross-species orthology-based marker selection is shown to be a powerful tool to quickly generate a comparative genetic map, which may speed up gene mapping and contribute to the understanding of genome evolution within the Solanaceae family.

## Background

*Solanum dulcamara* (bittersweet, climbing nightshade) is an allogamous diploid (2*n* = 2*x* = 24) species with a genome size of ~ 780 Mb [1]. It is one of the few *Solanaceae* species native to Europe, although it has been widely naturalised around the world (e.g. North America, Asia). *S. dulcamara* is placed in the clade Dulcamaroid, one of the 13 well-supported monophyletic clades in the *Solanum* section. The Dulcamaroid clade is closely related to the Morelloid clade, which includes *S. nigrum,* also native to Eurasia and considered as a weed of arable fields, and next to the Potato clade which includes species of economic importance such as *S. tuberosum* (potato) and *S. lycopersicum* (tomato) [2].

**Figure 1:**
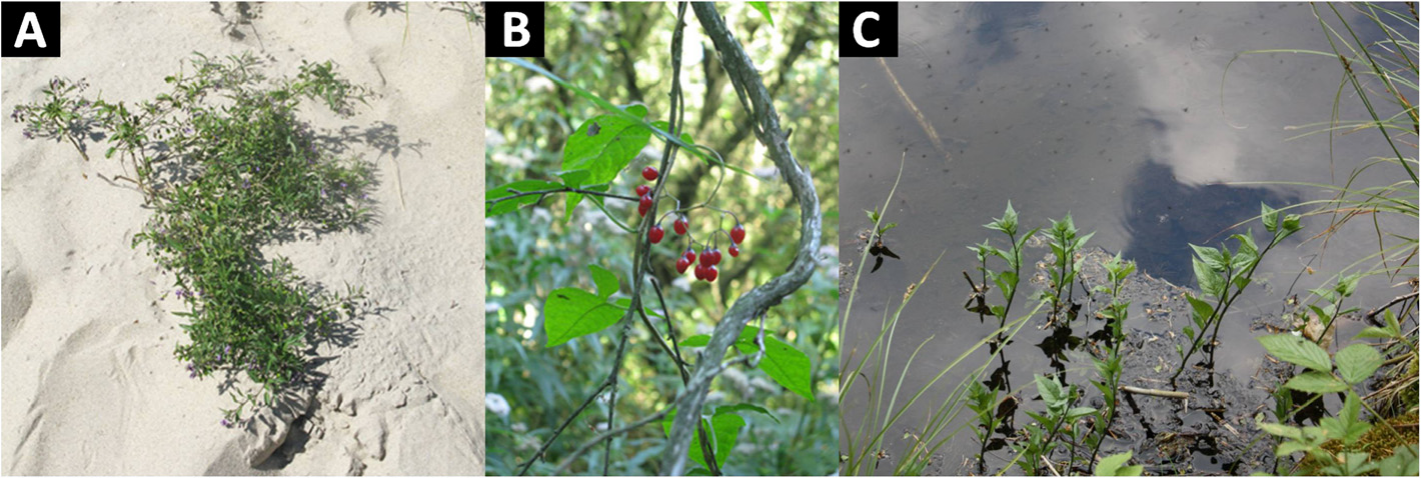
***S. dulcamara *phenotypes in different habitats.***S. dulcamara* growing as a creeping plant in a dry and open dune environment (**A**) and as a climbing plant in a moist and shaded forest undergrowth environment (**B**) and growing with its roots permanently flooded in a wet environment (**C**).

Bittersweet is adapted to grow in a wide range of ecological niches, from wet habitats such as riverbanks, lake shores and irrigation ditches to dry plains and dunes [3,4] (Figure 1). It is able to produce different phenotypes under different environmental conditions and in this respect it could represent a good model to study and explore the molecular bases of acclimation and adaptation. It has been demonstrated that *S. dulcamara* serves as an alternative host for many agriculturally important pathogens, such as *Ralstonia solanacearum*[5,6] and *Phytophthora infestans*[7] and pests, such as the Colorado Potato Beetle (*Leptinotarsa decemlineata*; T. Golas, personal observation). For this reason attempts were made to eradicate it from potato cultivation areas and beyond [8]. On the other hand, as a host it can represent a European source of resistance genes for *Solanum* breeding, as alternative to the currently used American sources. We have recently reported on the identification of two resistance loci to *Phytophthora infestans*, *Rpi-dlc1* and *Rpi-dlc2*, which are located on chromosome 9 and 10, respectively [9,10].

In spite of its important ecological role and potential to provide genetic resources for plant breeding, genomic research tools are lacking for *S. dulcamara* and only a very small number of nucleotide sequences (123) are currently available in GenBank [11]. Availability of a near-complete transcriptome, especially in combination with comparative genomics approaches and information transfer from related species with more genomics data can have a remarkable impact on the in-depth characterization of a species. Combining data and knowledge from the potato and tomato genome sequencing projects [12,13] with a *de-novo* RNAseq-based *S. dulcamara* transcriptome would thus be a powerful and valuable approach to speed up research on and exploitation of *S. dulcamara*.

We here present a deep sampling of the *S. dulcamara* transcriptome and first assessment of its complexity. The transcriptome enabled development of SSR and SNP markers, of which the latter were used to generate the first genetic map of *S. dulcamara*. This map was compared to the maps of tomato, potato and eggplant in order to elucidate chromosomal evolution within the genus and to contribute to future gene mapping efforts.

## Results and discussion

### *De novo* transcriptome assembly

**Figure 2:**
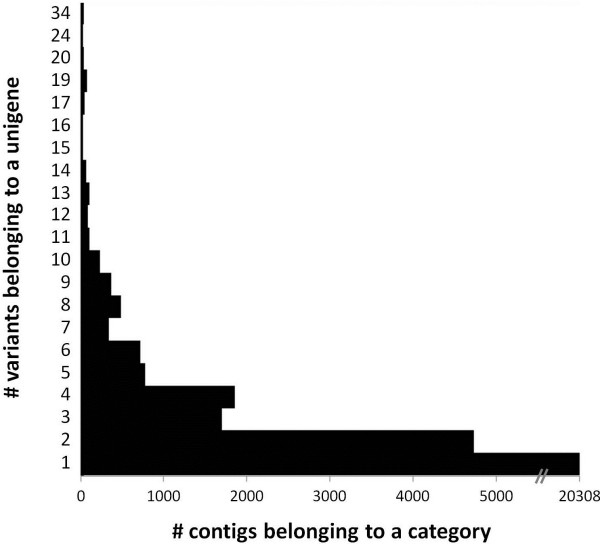
**Description of unigene cluster sizes.** The distribution of all contigs over categories of unigenes with single or multiple variant is plotted.

Short reads from seventeen different *S. dulcamara* cDNA libraries that were sequenced using either Roche GS-FLX or Illumina HiSeq2000 sequencing technologies (Table 1; Additional file 1: Table S1) were combined to build *de novo* a consensus transcriptome using the Trinity package [14]. This resulted in an assembly of 32,157 contigs of more than 500 nts in size, with an average length of 1,346 nts (Table 2; Additional file 1: Table S2). The dataset encompasses 24,193 unigenes, of which 3,885 are clusters with multiple variants (Table 2; Figure 2). These variants are expected to comprise allelic variants, splice variants, nearly-identical paralogs or mis-assemblies. The sequences of all contigs are available at the Sol Genomics Network website (http://ftp.solgenomics.net/unigene_builds/single_species_assemblies/Solanum_dulcamara/).

**Table 1:** **Summary of *S. dulcamara *cDNA RNAseq data sets used for assembly**

**Library**	**Sequencing method**	**# Reads (raw)**	**# Reads (high quality)**	**Avg read length (high quality)**
Mixed^1^	GS-FLX titanium, single end, random primed, normalised	799,937	786,113	297
Mixed^1^	GS-FLX titanium, single end, 3’ primed, normalised	568,762	558,729	312
Leaves^1^	HiSeq2000, single end, random primed, 100bp, normalised	127,318,285	107,557,660	96
Stem & primoridia^1^	HiSeq2000, single end, random primed, 50bp	516,292,741	471,046,908	50

^1^ See Additional file 1: Table S1 for details.

**Table 2:** **Statistics of the *de novo S. dulcamara *transcriptome assembly**

	
Total # of contigs	32,157
Total # of unigenes	24,193
# consisting of single sequence	20,308
# consisting of multiple variants	3,885
Total sequence length (nt)	43,277,997
Average contig length (nt)	1346
Minimum contig length (nt)	501
Maximum contig length (nt)	15047
Median contig length (nt)	1043

### Functional annotation

#### BLAST annotation

To attach biological information to each contig, a multi-step annotation workflow was designed (see Additional file 1; Table S2). First, sequence similarity search with BLASTx was performed against all tomato, potato and Arabidopsis predicted proteins as well as the UniProtKB/Swiss-Prot sequence set [15]. According to this analysis, 85% of the contigs presented at least one match at an E-value of e^-10^. No more than 47 contigs (0.15%) were found to have matches only to the UniProtKB/Swiss-Prot database, of which 30 were similar to sequences from viruses. Of these, 24 represented RNA replication and coat proteins from the potato virus M (PVM). This is in agreement with earlier findings of PVM in *S. dulcamara*[16,17], confirming it may serve as a reservoir for the virus from which it could move into potato. The remaining 17 contigs (0.05%) had significant matches to proteins from a wide spectrum of source organisms (from bacteria to human), and should be considered contaminations of the samples. Second, all the contigs that did not match any protein (4,829) were searched against the GenBank nucleotide non-redundant database with BLASTn. 1,913 contigs had correspondence to entries in the database at an e-value of e^-10^. Most of the first hits (88%) were sequences coming from Solanaceae species, with tomato the most represented. These sequences most likely represent UTRs or as yet un-annotated protein coding loci. The remaining sequences were similar to nuclear genes in GenBank (5%), mitochondrial DNA (4%), plastid DNA (1%) or ncRNAs, repetitive elements and sequences annotated as genomic markers (2%). Finally, 2,916 contigs, equal to ~9% of the assembled transcriptome, had no significant match in protein and nucleotide databases. The contigs in this dataset may encode for novel proteins, represent non-conserved UTR regions or are mis-assemblies.

#### Gene ontology and KEGG ortholog annotation

In order to describe gene functions in a standard and controlled vocabulary, we used the Blast2GO suite [18]. InterProScan searches were used to identify conserved protein domains in the *S. dulcamara* transcriptome and showed that 16,483 contigs (51.26%) had matches to conserved protein domains (Additional file 1: Table S2). Mapping of the InterPro entries to gene ontology (GO) terms resulted in the assignment of 33,008 GO terms to 12,637 contigs (Additional file 1: Table S2). The 32,157 *S. dulcamara* contigs were also analysed with the KEGG Automatic Annotation Server (KAAS) [19] to detect KEGG orthologs (KO). 5,283 *S. dulcamara* contigs representing KOs were identified (2,892 unique KO terms) (see Additional file 1: Table S2). Furthermore, 2,554 EC numbers could be associated to *S. dulcamara* contigs via the KO terms, resulting in the identification of 496 oxidoreductases, 868 transferases, 689 hydrolases, 152 lyases, 123 isomerases and 217 ligases.

All data combine to a high quality, thoroughly annotated draft of the *S. dulcamara* transcriptome.

### Comparison of protein family structure between *S. dulcamara* and other plant species

Multi-species transcriptome comparison may be used in order to identify orthologous gene groups, measure changes in the size of protein-coding gene families, study gene family evolution and detect taxonomically restricted sequences (i.e. species-specific or genus-specific sequences).

#### ORF/protein prediction

**Figure 3:**
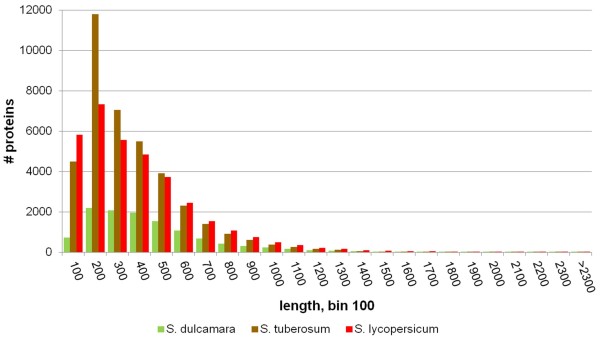
**Length distribution of proteins from *S. dulcamara *(bittersweet), *S. lycopersicum *(tomato) and *S. tuberosum *(potato).**

To be able to compare protein family structure between *S. dulcamara* and other plant species we first predicted the ORFs and protein sequences encoded by the *S. dulcamara* contigs. ESTScan [20] of the 32,157 contigs indicated that 26,696 contigs (~83%) contain putative coding sequences that could be translated into proteins. This is very similar to the percentage of contigs predicted to be protein coding by BLASTx (see above), with the slightly higher percentage of the latter probably explained by the fact that BLASTx better tolerates sequencing errors that result in frame shifts and premature stop codons than ESTScan. In total, 11,760 full-length proteins (i.e. containing a putative start and stop codon) and 14,936 truncated proteins (from partial coding sequences) were identified (Table 3). To confirm the reliability of the ESTScan prediction we carried out BLASTp searches of the predicted proteins against the tomato, potato and Arabidopsis protein complement. About 95% (25,418) of the *S. dulcamara* proteins had a significant match in at least one of these protein databases. Comparison of the BLASTp results with the BLASTx results of the same contigs revealed that in 99.9% of the cases, the best hit was identical. As a measure of the quality of our assembly, we also compared the size distribution of the subset of *S. dulcamara* full-length proteins to the length distribution of the proteins encoded in the genomes of tomato (34,727) and potato (39,031), the two *Solanum* species for which a full genome sequence was published recently [12,13]. Although the number of *S. dulcamara* full-length proteins is three to four times smaller than the number of proteins in the tomato and potato genome, protein size in the three datasets shows a similar log-normal distribution (Figure 3). Together, these results support the reliability of the assembly and the predicted protein data set.

**Table 3:** Results of the ORF prediction analysis

**Items**	**# contigs**
complete ORF	11,760
5'truncated ORF^1^	8,608
3' truncated ORF^2^	3,405
5'and 3' truncated ORF^3^	2,923
no good ORF^4^	5,461
Total	32,157

^1^ ORFs lacking the initiating ATG codon, but including a termination triplet.

^2^ ORFs including the ATG start codon, but lacking the stop codon.

^3^ ORFs having neither start nor stop codon.

^4^ Transcripts showing interspersed stop codons.

#### OrthoMCL clustering

**Figure 4:**
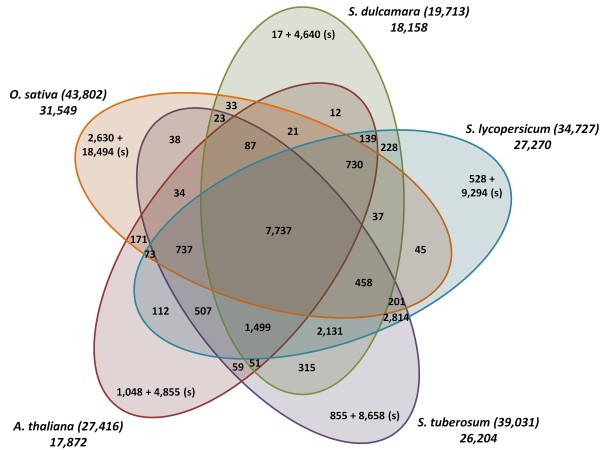
**Venn diagram describing the distribution of orthologous gene groups and singletons (s) identified by clustering 164,689 protein sequences from five different plant species using OrthoMCL.** For each species the total number of proteins sequences that were used as input (between brackets) and the total number of ortholog groups plus singletons are indicated.

Orthologous gene groups were identified using orthoMCL [21]. The analysis included protein datasets from *S. dulcamara,* from the related *Solanum* species tomato and potato, as well as from the two model plant species Arabidopsis and rice. As the input for *S. dulcamara* we used the partial and full-length proteins predicted by ESTScan. To ensure that each locus was represented only once in the orthologous gene group analysis, only the longest predicted protein from each variant cluster was used. Similarly for the other species, only the longest protein variant encoded by a locus was used. A total of 164,689 protein sequences from the five species were clustered into 23,370 ortholog groups (see Additional file 1: Table S3). A consensus annotation was automatically assigned to each group based on the frequency (greater or equal to 0.33) of the most prevalent InterPro entry list. In case the threshold criterion was not met, the combination of the two most frequent InterPro entry lists was used. In Figure 4, the number of orthologous and putative species-unique gene groups is shown. Of the 19,713 proteins from *S. dulcamara,* 15,073 were placed in a total of 13,518 gene groups with multiple members and 4,640 were not grouped and defined as species-specific singletons. As expected, a large part of the *S. dulcamara* gene groups (7,737) contained orthologs from all other species, thus representing genes that are highly conserved in flowering plants. High sequence conservation and high gene expression have been suggested to correlate [22-24], which may explain why the RNAseq-based *S. dulcamara* transcriptome has a slight bias towards highly conserved gene groups (43%), compared to the transcriptomes of tomato (31%) and potato (32%), which were derived from whole-genome sequencing. In *S. dulcamara*, as in the other species, many genes were species specific: 17 gene groups and 4,640 singletons.

#### Enrichment analysis

In order to understand which molecular functions were over-represented in the *S. dulcamara* specific set, we performed a GO enrichment analysis compared to all *S. dulcamara* proteins used for the OrthoMCL clustering. The analysis showed that genes associated with the molecular function terms “kinase activity” and “transporter activity” were most significantly overrepresented (Table 4), suggesting that these type of genes have evolved relatively fast in *S. dulcamara*. When looking at the *S. dulcamara* genes common only to either or both of the other two *Solanum* species (i.e. not to Arabidopsis and rice), molecular functions associated to regulation of gene expression and regulation of protein activity were enriched (Table 4). Again, this suggests that *S. dulcamara* genes involved in regulatory processes evolved relatively rapidly. Because the *S. dulcamara* transcriptome is not complete and nearly-identical paralogs are likely to collapse into variant clusters in the assembly step, possible expansions and contractions of gene families need further study.

**Table 4:** Enrichment analysis of GO molecular function terms in selected OrthoMCL subgroups when compared to the whole dataset

**GO.ID**	**Term**	**Annotated**	**Significant**	**Expected**	**p-value**
***S. dulcamara *specific**					
GO:0016301	kinase activity	1004	159	126.25	0.00086
GO:0016772	transferase activity. transferring phosphorus-containing groups	1004	159	126.25	0.00086
GO:0005215	transporter activity	534	89	67.15	0.00287
GO:0016740	transferase activity	1737	243	218.42	0.02998
***Solanum *specific**					
GO:0003677	DNA binding	1063	159	73.97	5.2E-22
GO:0001071	nucleic acid binding transcription factor activity	363	79	25.26	1.5E-20
GO:0003700	sequence-specific DNA binding transcription factor activity	363	79	25.26	1.5E-20
GO:0003676	nucleic acid binding	2017	209	140.36	1.6E-10
GO:0005515	protein binding	2827	242	196.72	8.5E-5
GO:0030234	enzyme regulator activity	165	21	11.48	0.0052
GO:0005488	Binding	8086	591	562.68	0.0091
***S. dulcamara *+ *S. lycopersicum *specific**					
GO:0003676	nucleic acid binding	2017	19	13.64	0.076
***S. dulcamara *+ *S. tuberosum *specific**					
GO:0030234	enzyme regulator activity	165	7	1.19	0.00018
GO:0004518	nuclease activity	103	3	0.74	0.03818
GO:0016788	hydrolase activity, acting on ester bonds	103	3	0.74	0.03818
GO:0003676	nucleic acid binding	2017	20	14.54	0.07853

### SSR analysis

#### In-silico *SSR prediction*

**Figure 5:**
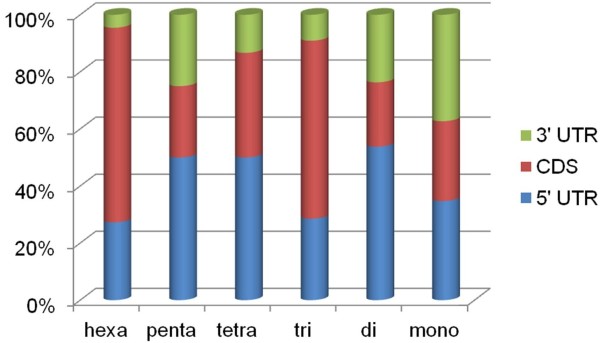
A stacked bar chart showing for each class of SSR the percentage located in the CDS, 5’ UTR and 3’UTR.

As a first effort aimed at the development and characterization of EST-based SSR markers in bittersweet, the *S. dulcamara* contigs were examined for the presence of SSR motifs using MISA (http://pgrc.ipk-gatersleben.de/misa/; Additional file 1: Table S4). A total of 6,029 SSRs were identified (Table 5). The frequency and the number of repeat units for each of the SSR motifs (except the mono-type repeats) are shown in Additional file 1: Table S5. With the exception of mono-type and compound repeats, the average length of the SSRs was ~16 nucleotides (range from 12 to 96 nucleotides). By exploiting the ORF/protein predictions from ESTScan, we also investigated the position of the SSRs along the transcripts (Figure 5). Tri-nucleotide as well as hexa-nucleotide repeats are preferentially located in the coding regions, while the other types are more frequent in UTRs. In particular, di-, tetra- and penta-nucleotide are preferentially located in 5' UTRs, whereas mono-nucleotide SSRs are equally distributed between 5' and 3' UTRs. These results are in agreement with observations in [25]. Using the Primer3 software [26], primer pairs to amplify each SSR were successfully designed for 4,233 transcripts and failed for 1,537 sequences (see Additional file 1: Table S4).

**Table 5:** **Summary of SSRs detected in the *S. dulcamara *transcriptome**

**Items**	**Counts**
Total number of identified SSRs:	6,029
Number of SSR containing sequences:	5,156
Number of sequences containing more than 1 SSR (c)	742
Mono-nucleotide (p1)	3,060
Di-nucleotide (p2)	1,163
Tri-nucleotide (p3)	1,733
Tetra-nucleotide (p4)	42
Penta-nucleotide (p5)	9
Hexa-nucleotide (p6)	22

#### In-vitro *SSR analysis*

To verify the usefulness of the SSRs as genetic markers and assess genetic diversity within a panel of seven *S. dulcamara* accessions, the list of putative SSRs for which primers were designed was first reduced to 344 by filtering out all mono-nucleotide SSRs and considering only those contigs that have a single orthologous counterpart in the tomato genome (see methods for criteria). Subsequently, 12 SSRs that seemed to be polymorphic in our short read libraries were amplified (see Additional file 1: Table S6). Capillary gel separation showed that the primer pairs for all but one SSR amplified fragments of around the expected size. All these microsatellites were polymorphic, having 2 to 4 alleles (Table 6). In all cases, heterozygous individuals were identified in our panel of seven plants, which is in agreement with the outcrossing biology of the species. Taken together, this shows that the set of SSRs and accompanying primer pairs is reliable and has potential use in the identification of marker-trait association as well as in the assessment of genetic diversity within *S. dulcamara* and between related species [27,28].

**Table 6:** Allele sizes of selected SSRs in various accessions

**Accession**	**Origin**	**SSR1 (c)**^**1**^	**SSR2 (p6)**^**1**^	**SSR3 (p2)**^**1**^	**SSR4 (p3)**^**1**^	**SSR5 (p4)**^**1**^	**SSR6 (p3)**^**1**^	**SSR7 (p2)**^**1**^	**SSR8 (p4)**^**1**^	**SSR9 (p3)**^**1**^	**SSR10 (p3)**^**1**^	**SSR12 (p3)**^**1**^
A54750069-1	UK	n.a.	219	209+211	151	269+277	269	244	173	272+275	226+232	196
944750001-2	UK	n.a.	219	211+213	148	269+277	269	248+250	165+173	275+281	232+235	190
A94750045	NL	231	213+219	207+209	148+151	269+277	260+269	244+248	165	275+281	226+232	193
A94750066	NL	237+240	213	207+209	151	269+277	260+269	244	173	275	226+232	n.a.
A94750135	NL	237	207+219	213	148+151	269	269+272	248	173	275+281	226+232	190+193
A94750168	NL	240	213+219	211+213	151	269	260	244	173	272+275	232	193+196
A6/005-1	A	n.a.	201+207	213	145+154	269	269+275	246+248	165+173	272+281	226+232	190+193
												
n. alleles		3	4	4	4	2	4	4	2	3	3	3
n. homozygous		3	3	2	4	3	3	4	5	1	1	3
n. heterozygous		1	4	5	3	4	4	3	2	6	6	3

^1^ SSR type (see Table 5).

*A*: Austria; *NL*: The Netherlands; *UK*: United Kingdom; *n.a.*: not available.

### Genetic map construction

*S. dulcamara* contains a number of traits, such as several pathogen resistances, which may be valuable for agricultural purposes. To speed up gene mapping efforts in this species we generated a genetic map that is anchored to the high-quality genetic/physical maps of tomato. In addition to providing practically useful information on synteny and co-linearity between these two species, such a map also offers insight into genome evolution in the genus *Solanum*.

#### SNP identification

For map construction, we first identified sequence polymorphisms between accessions A54750069-1 and 944750001-2 for which an F_1_ population was available [10] (Additional file 1: Table S1). By independently mapping the reads of the two parent genotypes to a subset of the contigs, 20,162 putative SNPs were identified (see methods for criteria). The observed transition:transversion ratio of 1.52 is very similar to what has been reported for SNP types in other plant species [29,30] (Additional file 1: Table S7).

#### Segregation analysis and map construction

**Figure 6:**
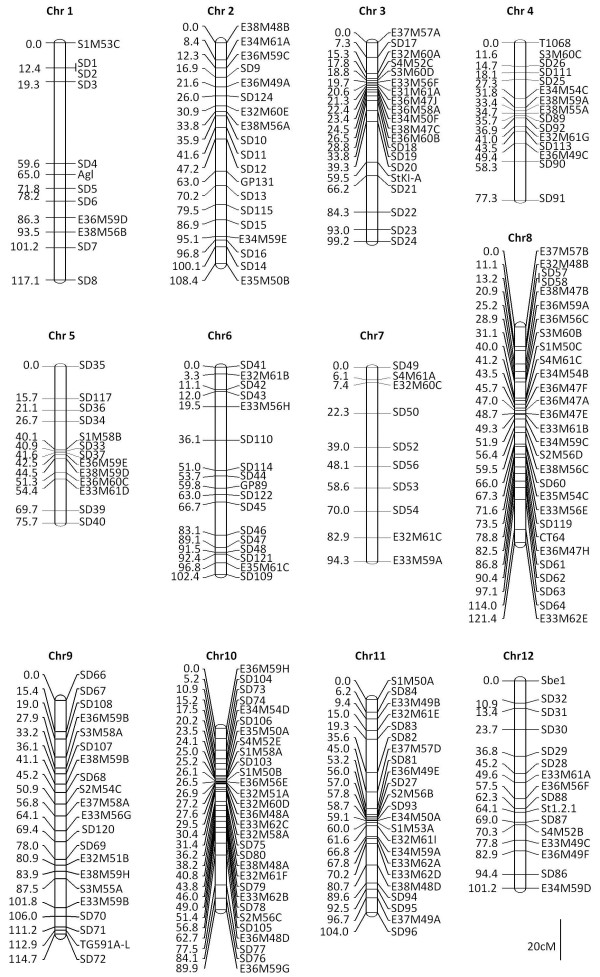
**A genetic map of *S. dulcamara*.** The map is based on 225 markers: 12 linkage groups (chromosomes) were identified. The genetic position (in cM) with respect to the first marker in the linkage group is indicated. Marker codes SD1 to SD96 refer to SNP markers analysed with KASPar assays, marker codes SD103 to SD122 refer to SNP markers analysed with CAPS assays, marker codes starting with E/S##M## refer to AFLP markers (see http://wheat.pw.usda.gov/ggpages/keygeneAFLPs.html for standard nomenclature). All other marker codes refer to published markers from potato.

**Figure 7:**
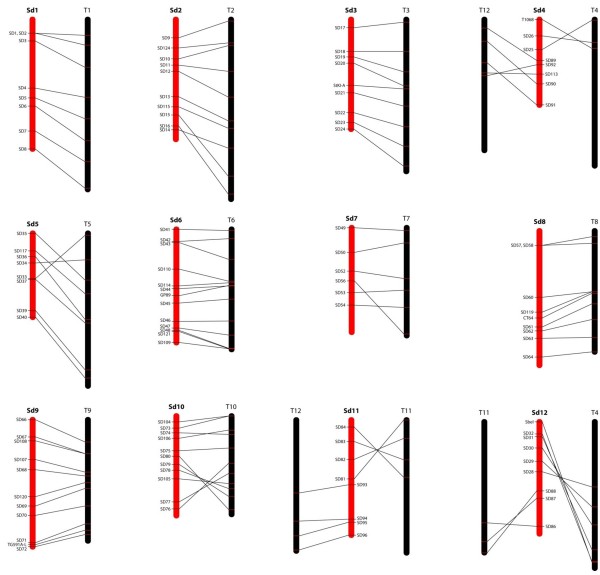
**Comparative analysis of the *S. dulcamara *and tomato chromosomes.**

A subset of SNPs was identified that was heterozygous in the female parent and homozygous in the male parent (see methods for criteria) and 96 of these were selected in such a way that the tomato orthologs of the corresponding contigs were distributed evenly over the tomato genome (Additional file 1: Tables S2 and S8). Segregation of 90 out of 96 selected SNP could successfully be determined in 94 F_1_ individuals, using KASPar assays (http://www.lgcgenomics.com/). In addition, segregation of 108 AFLP and 27 CAPS markers was analysed in the same individuals. Linkage analysis using JoinMap [31] revealed the existence of 12 linkage groups, in agreement with the haploid chromosome number of the species. The resulting linkage groups ranged from 76 to 121 cM in size and harboured 10 to 30 markers each (Figure 6; Additional file 1: Table S9). Synteny and co-linearity with tomato was studied using marker orthology (Figure 7; Additional file 1: Table S8). To better understand chromosome evolution in the Solanaceae, the genetic map was subsequently compared to the integrative maps of tomato (*S. lycopersicum*), potato (*S. tuberosum*), eggplant (*S. melongena*), pepper (*Capsicum annuum*), tobacco (*Nicotiana*) and their deduced common ancestors, as presented by Wu and Tanksley [32] (Additional file 2: Figure S1).

#### Chromosomes co-linear with tomato

*S. dulcamara* (Sd) chromosomes 1, 3, 6, 8 and 9 are syntenic and fully co-linear with their respective tomato (T) counterparts. Co-linearity of Sd and T chromosomes suggests that their arrangement represents the ancestral tomato/bittersweet arrangement. In case of Sd1/T1, Sd3/T3 and Sd8/T8 this is in agreement with the suggestion that also the more ancient common ancestor of tomato/bittersweet and eggplant (the ancestral *Solanum*) is likely to have had these arrangements [32].

In case of chromosome 6, tomato and potato contain two small inversions, located at the top and bottom ends of the chromosome, when compared to eggplant and their common *Solanum* ancestor [32]. Whether these occurred before or after the deviation of bittersweet could not be evaluated, because only a single and no markers are present in those regions, respectively.

The finding that Sd9 is fully co-linear to T9 is surprising, because Wu and Tanksley [32] suggested that tomato harbours an inversion that arose after the split from potato. More detailed examination of this region in potato, however, suggests this inversion is also present, but was followed-up by a second inversion, unique to potato, largely restoring the eggplant like arrangement [33]. Thus, tomato and bittersweet have retained the arrangement ancestral to potato, in which there is a unique inversion.

#### Chromosomes with inversions compared to tomato

*S. dulcamara* chromosomes 2, 5, 7 and 10 are syntenic with their corresponding tomato counterparts, but show intra-chromosomal rearrangements (inversions). The difference between Sd2 and T2 is most easily explained by two subsequent inversions in the same region. The inversion between SD14 and SD16 (taking tomato as the reference; Figure 7) overlaps with an inversion seen in potato and eggplant and may thus represent the ancestral *Solanum* arrangement [32]. The second inversion, between SD15 and SD16 (Figure 7) is similar to one seen in eggplant. Wu and Tanksley [32] argued that this inversion is not present in the more distant relative pepper, and is thus eggplant-specific. Taking into consideration our data, however, the inversion might have been present in the common *Solanum* ancestor and reversed in the tomato/potato lineage, or alternatively, occurred independently in bittersweet and eggplant. Either scenario would imply repeated use of the breakpoint. However, it should be noted that detailed examination of this chromosome segment has shown that it has undergone complex rearrangements, making conclusive interpretations difficult [34].

Sd5 shows a double inversion with respect to T5, while having the same gene content. The large inversion from SD33 to SD36 (taking tomato as the reference; Figure 7) is tomato-specific, as the bittersweet structure is similar to that in potato and the ancestor of *Solanum*. The second, nested inversion between SD35 and SD36 (Figure 7) is shared with the more distant relative pepper. Wu and Tanksley [32] argued that this inversion was not easily recognisable in eggplant, which has complex re-arrangements in this chromosomal region, and was thus already absent in the ancient *Solanum*, implying a reversal in bittersweet. A more parsimonious explanation would be that the bittersweet/pepper marker order is ancestral so *Solanum*, and that the SD35/SD36 region diverged independently in eggplant and tomato/potato lineages. The translocation of the bottom half of T5 and potato (Pt) chromosome 5 with respect to eggplant and their last common ancestor must have occurred early after the split from eggplant, as it is already present in bittersweet.

Sd7 has the same gene content as T7, but shows two inversions relative to tomato, potato and eggplant, which thus occurred in the bittersweet lineage, after its separation from the tomato/potato lineage.

Sd10 contains the same genes as T10, with two inversions. The large long-arm inversion is tomato-specific; the arrangement in bittersweet being ancestral to the genus *Solanum*. The origin of the smaller inversion at the top half is hard to deduce, because a similar rearrangement can be seen in eggplant and its more distant relative tobacco, but not in the intermediate relative pepper. Irrespective of the evolutionary order of the rearrangements, this means inversions with similar breakpoints have occurred multiple times in this region.

#### Chromosomes showing translocations relative to tomato

The gene content of tomato chromosomes 4, 11 and 12 is represented by *S. dulcamara* chromosomes 4, 11 and 12, but with a different inter-chromosomal arrangement. Sd4 consists of the upper part of T4 and the upper part of T12. Roughly the same upper part of T4 associates with parts of T11 in eggplant and the more distant relatives pepper and tobacco [32], suggesting that both, the arrangement in tomato-potato and in bittersweet are derived within their respective lineages. Likewise, the same upper part of T12 associates with parts of T5, T3/T9 and T6 in eggplant, pepper and tobacco, respectively. Because of this complexity, it is impossible to deduce ancestral chromosomal arrangements and the origin of the inversions between bittersweet and tomato. Repeated usage of the translocation breakpoints shows that these chromosomes are unstable over evolutionary time.

The top half of Sd11 has the same gene content as T11, but is inverted. The same inversion is not only seen in potato, but also in eggplant, pepper and tobacco, although the latter was not indicated by Wu and Tanksley [32]. This orientation thus is ancestral to *Solanum*. However, while the top of T11 is associated with the bottom part of T12 in bittersweet, the more ancestral association is with the upper part of T4. The bottom part of T12, in turn, has been associated to parts of T5, T4/T3/T9 and T8 in eggplant, pepper and tobacco, respectively, again indicating repeated usage of a region as a translocation breakpoint.

Sd12 consists of the lower half of T4 and the bottom of half of T11, the latter being inverted in orientation. This inversion can also be seen in eggplant and pepper [32], and is therefore ancestral to *Solanum*. Again, these chromosomal segments have been associated to various different fragments during the evolution of the Solanaceae.

## Conclusion

We present a variety of genomics resources for the non-model species *S. dulcamara* and demonstrate their use for functional, genetic and comparative analyses. The large-scale characterization of the bittersweet transcriptome provides a first catalogue of the *S. dulcamara* gene repertoire and allowed SNPs and SSRs to be identified and successfully used as genetic markers for the generation of a linkage map and the analysis of genetic diversity, respectively. We show that molecular markers derived from transcribed regions can be anchored to the genomes of related species for map comparison. Such information is very useful for gene mapping efforts, as we recently showed for mapping of the *Rpi-dlc2* locus, which is located near the inversion breakpoint on chromosome 10, in comparison to tomato [10]. The observed chromosome inversions as deduced from the genetic map concur well with previously published data from other Solanaceae and support the position of *S. dulcamara* in the tomato/potato clade (“Clade I”) [2]. Furthermore, the data sustain the notion that certain chromosomal regions are more likely to serve as inversion and translocation breakpoints. For chromosome Sd4, -11 and -12 we report a new chromosome composition of segments that in other species are also associated with translocations. For future research, the *S. dulcamara* transcriptome will serve as a reference for RNAseq gene expression profiling and be used to facilitate functional genomics studies. This is crucial to the identification of key regulators of important biological phenomena, such as adaptation to different environmental conditions and responses to biotic stressors. Together, this will allow us not only to target genes underlying important agronomic traits, but also help us understand and exploit the unique biology of this species.

## Methods

### Plant material

*S. dulcamara* material used to generate mRNA samples for RNAseq is described in Additional file 1: Table S1. Material used to test SSRs was provided by Dr Janny Peters (Radboud University Nijmegen, The Netherlands). The segregating population used for map construction (code 05-150) was derived from a cross between accession A54750069-1 and 944750001-2 [10]. All plants were cultivated in standard greenhouse conditions as described in [10], unless indicated otherwise.

### RNA extraction and sequencing

Total RNA was isolated using Trizol (for the “Mixed” and “Leaves” libraries, see Table 1) or the Plant RNeasy kit (Invitrogen; for the “Stem & primordia” library, see Table 1) and treated with DNase. In case of the “Mixed” libraries, mRNA was purified and duplex-specific-nuclease-normalized cDNA samples were prepared and sequenced by Eurofins MWG Operon (Ebersberg, Germany) on the Roche GS-FLX platform. In case of the “Leaves” library, mRNA was purified and duplex-specific-nuclease-normalized cDNA samples were prepared and sequenced by Fasteris SA (Geneva, Switzerland). For the “Stem & primordia” library, mRNA was purified and cDNA samples were prepared and sequenced by Fasteris SA without prior normalisation.

### *De novo* transcriptome assembly

Raw read filtering based on quality values and length was performed with the ‘Trim sequences’ algorithm in CLC Genomics Workbench v4.7.1 (CLCBio, Aarhus, Denmark). Default settings were used and low quality sequences (limit=0.05) and sequences no longer than 50 nts were removed. Although the assembler algorithm discarded low coverage k-mers, the raw reads were error corrected in order to speed up the assembly process. Therefore all sequence data except the Roche GS-FLX data was base-error-corrected with decGPU version 1.06 [35]. DecGPU was run with default settings. The decGPU algorithm output consisted of error free reads, fixed reads and discarded reads. For the assembly both error free and fixed reads were used. The decGPU process discarded 66M sequences (11% of total Illumina input sequences). All samples where pooled, both Roche GS-FLX and Illumina sets, and assembled using the *de novo* transcriptome assembler Trinity version 2011-10-29 (http://trinityrnaseq.sourceforge.net/) [14]. The Trinity assembly was run with a default fixed k-mer length of 25, minimal contig length of 500 bp, minimal k-mer coverage of 2 and a butterfly heap space size of 50GB.

### ORF identification and functional annotation

Automated annotation was performed by BLASTp and BLASTx searches (e-value < e^-10^) against the *S. lycopersicum* (version iTAG 2.3 proteins), *S. tuberosum* (version 3.4), *A. thaliana* protein complement (version TAIR 10 pep) and the UniProtKB/Swiss-Prot database (release 2012_02)*.* In addition, BLASTn searches (e-value < e^-10^) against the nucleotide non-redundant database (GenBank, release March 2011) were carried out. The Blast2GO suite (version 2.5.0) [18] was used to identify InterPro entries that were mapped to GO terms. KAAS [19] was used to assign KO (KEGG orthologs) terms (representative gene data set for eukaryotes + plants) to *S. dulcamara* transcripts. The BBH (bi-directional best hit) option was used to map KO terms onto KEGG pathways, using the same program.

### Identification and annotation of orthologous gene groups

ESTScan [20] was used to predict ORFs in the *S. dulcamara* transcriptome using the default *Arabidopsis thaliana* training matrix for peptide prediction. OrthoMCL (version 2.0.2) [21] was used to identify gene family groups among *S. dulcamara* (19,713), *S. lycopersicum* (34,727), *S. tuberosum* (39,031), *A. thaliana* (27,416), *O. sativa* (43,802). Enclosed within brackets, is reported the number of proteins used as input data, after removing all but the longest protein sequence in case of splice variants. All the resulting sequences were merged into a single FASTA file and all-versus-all comparisons were performed using BLASTp (e-value < e^-5^). For the MCL clustering algorithm we used an inflation value (-I) of 1.5 (OrthoMCL default). Consensus annotation of each gene group was automatically assigned based on of the most frequent InterPro entry list (frequency ≥ 0.33). In case the threshold criterion was not satisfied, the combination of the two most frequent InterPro entry lists was used. In case of Arabidopsis, rice and tomato we exploited the already available nterPro annotations (http://ftp.arabidopsis.org/home/tair/Genes/TAIR10_genome_release/TAIR10_functional_descriptions; http://rapdb.dna.affrc.go.jp/download/archive/irgsp1/IRGSP-1.0_representative_2012-05-28.tar.gz; http://ftp.solgenomics.net/tomato_genome/annotation/ITAG2.3_release/ITAG2.3_desc_and_GO.csv). In contrast, because no InterPro annotation is available at http://potatogenomics.plantbiology.msu.edu/index.html, we identified the InterPro protein domains within the potato sequence collection using the Blast2GO suite. The GO term enrichment analysis was performed using TopGO package (v2.8) from Bioconductor (http://www.bioconductor.org/packages/2.12/bioc/html/topGO.html). Fisher's exact test (p-value < 0.01) was used to identify the over-represented GO terms.

### SSR identification and analysis

The SSR search tool MISA (MIcroSAtellite identification tool; http://pgrc.ipk-gatersleben.de/misa/) was used to identify and localize single or multiple stretches of microsatellite motifs. Research criteria include a minimum of 10 in case of mononucleotide and a minimum of 4 repetitive units in case of 2-, 3-, 4-, 5-, 6- unit repeats. Primer pairs flanking the microsatellite loci were automatically designed using Perl scripts provided with MISA (http://pgrc.ipk-gatersleben.de/misa/primer3.html) in combination with the software Primer3 (http://www.broadinstitute.org/genome_software/other/primer3.html) [26].

Genomic DNA was isolated from seven individuals using a standard CTAB method [36,37]. PCR amplifications were performed in 20 μL volume and the reaction mixture contained 16 ng of genomic DNA, 100 μM of each primer, 400 μM of dNTPs each, 0.5 units of DreamTaq Polymerase (Fermentas), 1× DreamTaq DNA polymerase buffer containing 20 mM MgCl_2_. PCR conditions consisted of an initial denaturation step at 95°C for 2 min, followed by 40 cycles of 95° C for 20 sec, 55°C for 30 sec, and 72°C for 20 sec and a final step at 72°C for 30 min. Forward primers were labelled with WellRED D2-PA fluorescent dye (Sigma-Aldrich). 1 μL of PCR product and 0.3 μL of GenomeLab DNA Size Standard 400 (Beckman Coulter) were diluted with 30 μL sample loading solution (Beckman Coulter) and electrophoresis was performed on the CEQ 8800XL Genetic Analysis System (Beckman Coulter). SSR locus allele sizes were determined using the Beckman CEQ fragment analysis software.

### SNP discovery and KASPar marker design

For SNP discovery, RNAseq sequence reads were first trimmed and mapped to the *S. dulcamara* transcriptome assembly using CLC Genomics Workbench v4.7.1 (CLCBio, Aarhus, Denmark). For trimming, low quality sequence (limit 0.05) and ambiguous nucleotides (if longer than 2 nucleotides) were removed and reads shorter than 50 nucleotides were discarded. As a template, we selected contigs with an average coverage of more than 20 and less than 10,000 reads. For read mapping, the minimal read coverage was set at 90% and the minimal alignment identity at 90%. Reads that could be mapped to multiple locations with the same score (repeats) were assigned randomly to one of these locations. SNP calling was done using an upgraded version of QualitySNP [38] (H. Nijveen, Wageningen University, The Netherlands, unpublished). To determine valid SNPs, the minimum similarity score per polymorphic site was set at 0.75 and the minimum similarity score of all polymorphic sites at 0.8, INDEL SNPs were marked as low quality and removal of low quality at sequence ends was disabled. For SNP selection, SNPs were taken as heterozygous in A54750069-1 when coverage was at least 10 reads and frequency of each allele was more than 30%. SNPs were taken as homozygous in 944750001-2 when coverage was at least 20 reads and the number of alternative allele reads was 0. Furthermore, the GC content of the 50bp flanking the SNP on either side was between 30 and 70% to aid in primer design. Primers for KASPar assays (http://ftp.solgenomics.net/tomato_genome/annotation/ITAG2.3_release/ITAG2.3_sgn_data.gff3) were designed by KBioscience (Hoddesdon, UK) and assays were performed according to the manufacturer’s protocol on a Fluidigm EP1 system (Fluidigm Corporation, San Francisco, CA). A BLASTp-based analysis was performed to identify probable orthologous pairs as reciprocal best hits between bittersweet and tomato. BLASTp hits with bit scores lower than 100 were filtered out. In addition, BLASTp searches were performed to identify near identical paralogs within the reference species tomato. In case the bit score of a paralogous pair was higher than the score of the corresponding orthologous pair, the corresponding tomato transcript was regarded as having in-paralogs (i.e. paralogs arising from duplication after speciation) and, unless paralogs were located not more than 10 loci apart, discarded from the analysis. The position of tomato genes on the genetic map was estimated through the identification of the closest 3’ and 5’ marker from the tomato Kazusa F2-2000 linkage map available at the SGN ftp site [39]. Physical positions were retrieved from a gff3 file that contains alignments of marker sequences to the tomato pseudo-molecules (http://solgenomics.net/itag/release/2.3/list_files/ITAG2.3_sgn_data.gff3).

### Genetic map construction and comparison

An *S. dulcamara* F_1_ population containing 94 individuals was screened with 328 SNP, CAPS and AFLP markers (Additional file 1: Tables S8 and S9). Markers with >75% missing data points, completely linked markers (except for SD2/SD58, used for anchoring to the tomato map) and markers showing a segregation ratio significantly different from 1:1 (χ^2^ test, p<0.005) (except for SD2/SD58, used for anchoring to the tomato map) were excluded prior to analysis. Regarding the KASPar assays, in one case no SNP was detected and in four cases the assay did not work properly. Matrix data was analysed using JoinMap v4.1 [31] with CP (cross-pollination) population type settings. For linkage analyses the regression and maximum-likelihood mapping algorithms were used, with Haldane’s mapping function. Markers with different relative positions on the two generated maps and markers that could not be assigned to a linkage group were rejected. The positions of 225 markers, as obtained by regression mapping, are presented here. Rooting of groups was done with LOD>4. For comparison of the *S. dulcamara* genetic map with those of other Solanaceae as produced by Wu and Tanksley [32], all markers were mapped to the tomato Kazusa F2-2000 linkage map. Three markers were excluded from the genetic map because they suggested singleton translocations that were not corroborated by neighbouring markers: Agl (*S. dulcamara* chr1, 65.0 cM, *S. lycopersicum* chr4, 36.5 cM), SD27 (*S. dulcamara* chr11, 57.0 cM, *S. lycopersicum* chr4, 37.4 cM) and SD122 (*S. dulcamara* chr6, 63.0 cM, *S. lycopersicum* chr2, 35.2 cM).

### Accession numbers

Raw sequence reads obtained from 454 and Illumina sequencing were submitted to the NCBI Short Read Archive (SRA, http://www.ncbi.nlm.nih.gov/sra) under the accessions SRP020226. The *S. dulcamara* contigs are available from the Sol Genomics Network website (http://ftp.solgenomics.net/unigene_builds/single_species_assemblies/Solanum_dulcamara/) and are included in the SGN BLAST search tool (http://solgenomics.net/tools/blast/index.pl?db_id=203).

## Competing interests

The authors declare that they have no competing interests.

## Authors’ contributions

JZ, TD and TG prepared samples for RNAseq. HG assembled the transcriptome and performed SNP discovery. NDA, AB and JB performed transcriptome analysis and annotation. NDA performed SSR identification and verification. JPN supervised the transcriptome assembly. TG, ND and EW build the genetic map. NDA, CM and IR designed the study, IR coordinated the study and NDA, JPN and IR wrote the manuscript. All authors have seen and agreed with the final version of the manuscript.

## Supplementary Material

Additional file 1: Table S1 Description of RNAseq libraries. **Table S2:** List of assembled contigs with BLAST annotation, InterPro entries, GO terms as well as KO terms and EC numbers associated to KEGG pathways. **Table S3:** Gene groups identified by the OrthoMCL analysis with number of members per species and including consensus annotation of each OrthoMCL gene group based on InterPro descriptions. Tot, total number of members; Sdu, S. dulcamara; Sly, S. lycopersicum; Stu, S. tuberosum; At, Arabidopsis thaliana; Osa, Oryza sativa. **Table S4:** Details of the in-silico identified SSRs and of the primer pairs successfully designed for their amplification. SSR types are abbreviated as described in Table 5. **Table S5:** Distribution and number of repeat unit of SSR motifs in the transcriptome. SSRs were classified considering the repeat motifs in both directions. SSR types are abbreviated as described in Table 5. **Table S6:** List of SSR markers that were experimentally assessed. SSR types are abbreviated as described in Table 5. **Table S7:** SNP statistics. **Table S8:** Description of the markers used for KASPar and CAPS genotyping. Genetic positions of anchored markers are indicated in S. dulcamara and tomato. **Table S9:** Statistics of markers in the genetic map. Click here for file

Additional file 2: Figure S1 Comparative maps of S. dulcamara and several other solanaceous species and the deduced genome arrangement (modified from [32]). K-2000 refers to the Kazusa F2-2000 genetic map; E-2000 refers to the Expen 2000 genetic map. For detailed legend to the figure, please see Additional file 3: Figure S3 in [32]. Click here for file
