# Supplementary material for: Genomic analysis of the native European Solanum species, S. dulcamara
Source: BMC Genomics. 2013 May 28;14:356. doi: 10.1186/1471-2164-14-356 (PMC3680029; doi:10.1186/1471-2164-14-356)
Supplement: Additional file 2: Figure S1 — Comparative maps of S. dulcamara and several other solanaceous species and the deduced genome arrangement (modified from [32]). K-2000 refers to the Kazusa F2-2000 genetic map; E-2000 refers to the Expen 2000 genetic map. For detailed legend to the figure, please see Additional file 3: Figure S3 in [32]. [file 1471-2164-14-356-S2.ppt]

## Slide 1
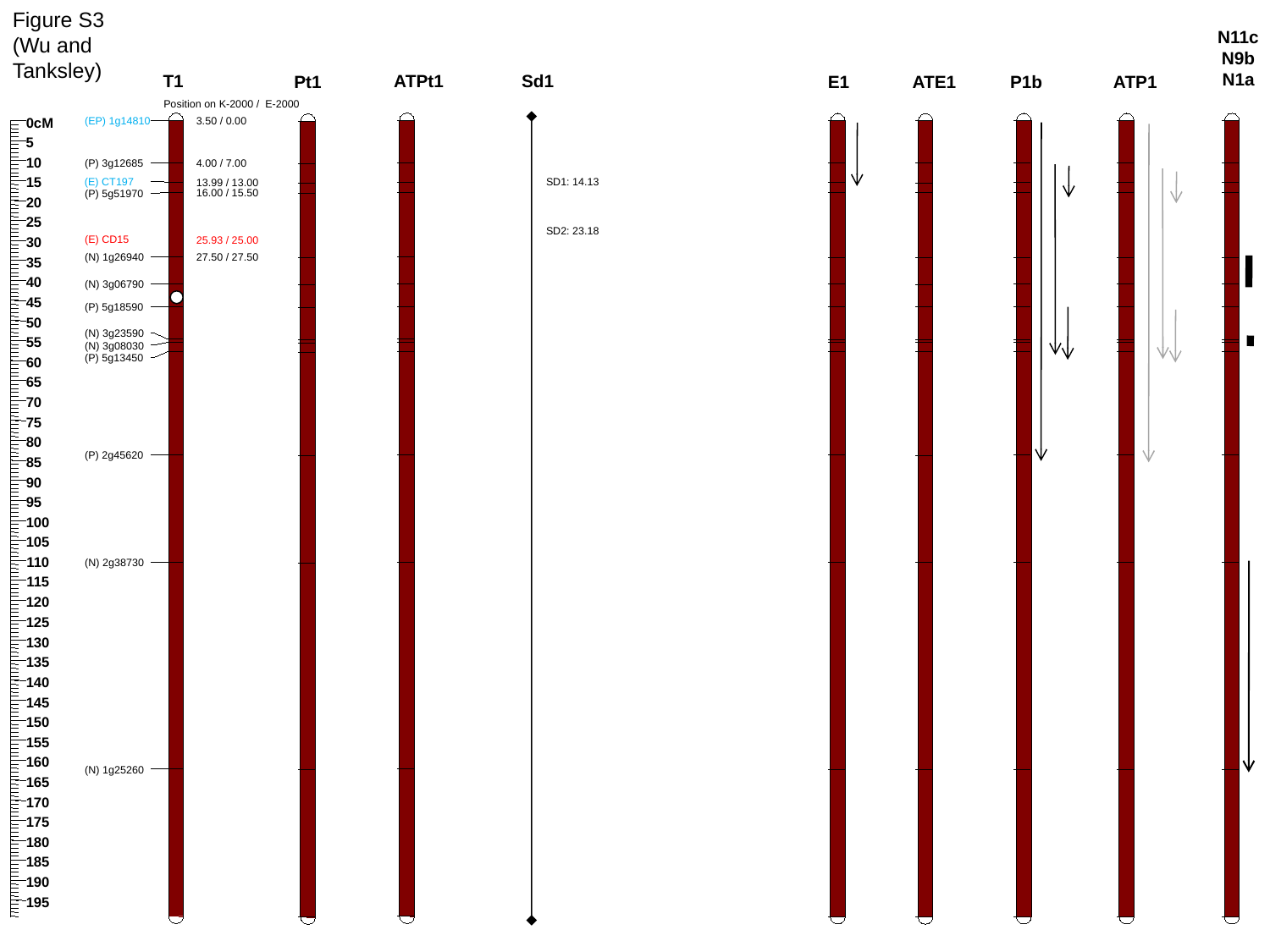

Figure S3
(Wu and Tanksley)
N11c
N9b
N1a
T1
(EP) 1g14810
(P) 3g12685
(E) CT197
(P) 5g51970
(N) 1g26940
(N) 3g06790
(P) 5g18590
(N) 3g23590
(N) 3g08030
(P) 5g13450
(P) 2g45620
(N) 2g38730
(N) 1g25260
ATPt1
Sd1
E1
P1b
ATP1
ATE1
Pt1
Position on K-2000 / E-2000
3.50 / 0.00
0cM
5
10
15
20
25
30
35
40
45
50
55
60
65
70
75
80
85
90
95
100
105
110
115
120
125
130
135
140
145
150
155
160
165
170
175
180
185
190
195
4.00 / 7.00
SD1: 14.13
13.99 / 13.00
16.00 / 15.50
SD2: 23.18
25.93 / 25.00
(E) CD15
27.50 / 27.50

## Slide 2
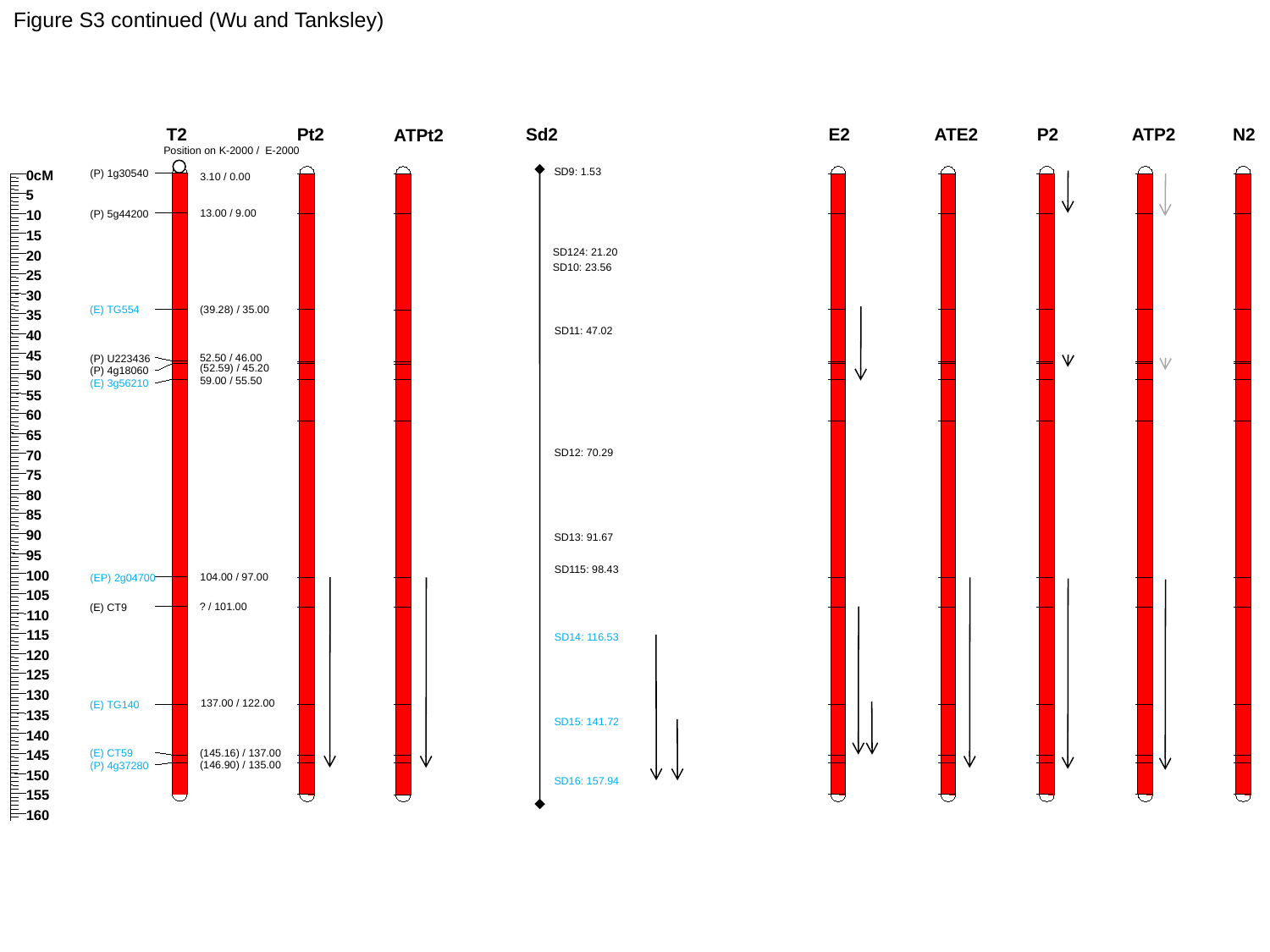

Figure S3 continued (Wu and Tanksley)
T2
(P) 1g30540
(P) 5g44200
(E) TG554
(P) U223436
(P) 4g18060
(E) 3g56210
(EP) 2g04700
(E) CT9
(E) TG140
(E) CT59
(P) 4g37280
Sd2
Pt2
E2
ATE2
P2
ATP2
N2
ATPt2
Position on K-2000 / E-2000
SD9: 1.53
3.10 / 0.00
0cM
5
10
15
20
25
30
35
40
45
50
55
60
65
70
75
80
85
90
95
100
105
110
115
120
125
130
135
140
145
150
155
160
13.00 / 9.00
SD124: 21.20
SD10: 23.56
(39.28) / 35.00
SD11: 47.02
52.50 / 46.00
(52.59) / 45.20
59.00 / 55.50
SD12: 70.29
SD13: 91.67
SD115: 98.43
104.00 / 97.00
? / 101.00
SD14: 116.53
137.00 / 122.00
SD15: 141.72
(145.16) / 137.00
(146.90) / 135.00
SD16: 157.94

## Slide 3
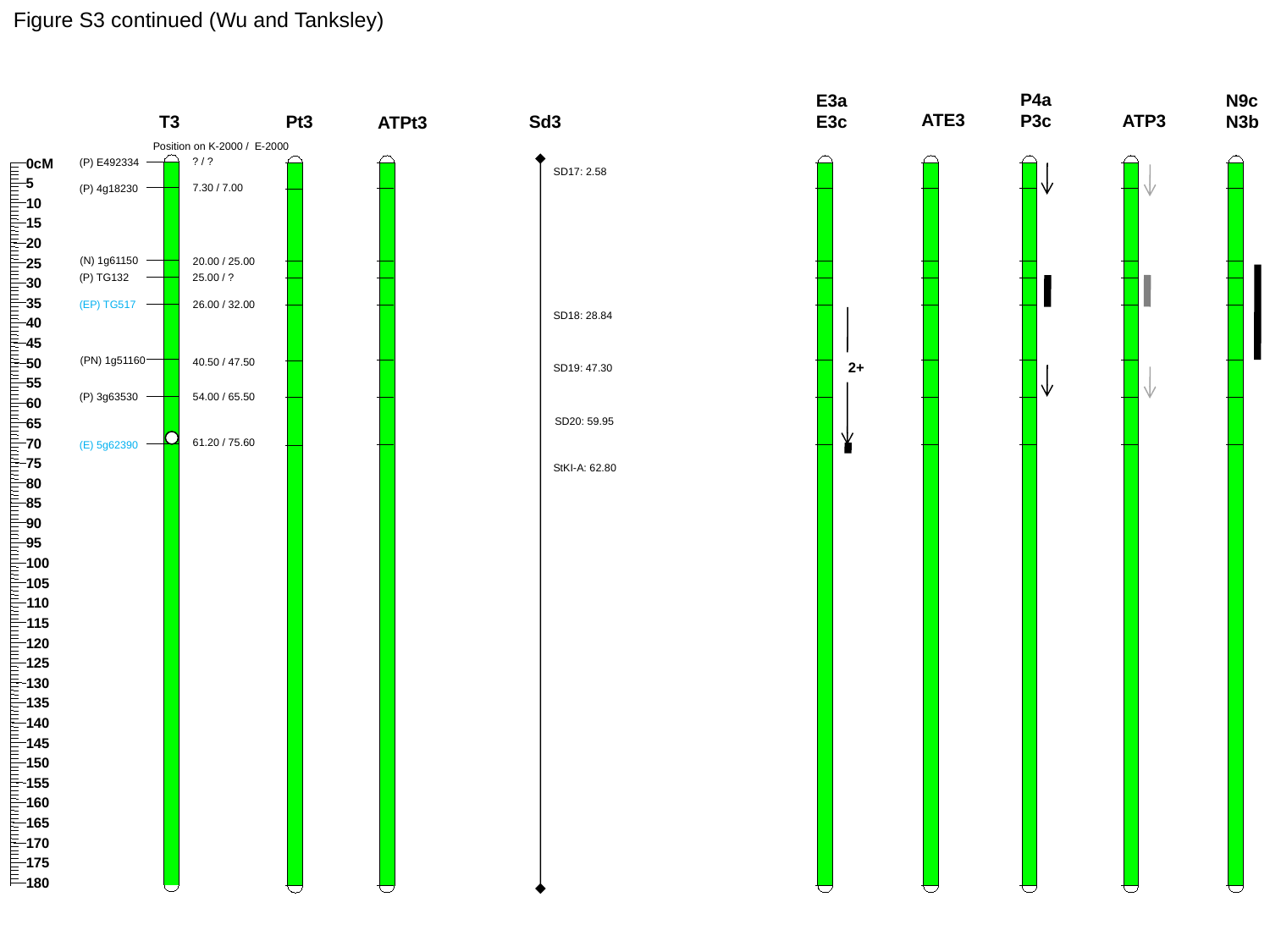

Figure S3 continued (Wu and Tanksley)
P4a
P3c
E3a
E3c
2+
N9c
N3b
ATE3
ATP3
T3
(P) E492334
(P) 4g18230
(N) 1g61150
(P) TG132
(EP) TG517
(PN) 1g51160
(P) 3g63530
(E) 5g62390
Pt3
Sd3
ATPt3
Position on K-2000 / E-2000
? / ?
0cM
5
10
15
20
25
30
35
40
45
50
55
60
65
70
75
80
85
90
95
100
105
110
115
120
125
130
135
140
145
150
155
160
165
170
175
180
SD17: 2.58
7.30 / 7.00
20.00 / 25.00
25.00 / ?
26.00 / 32.00
SD18: 28.84
40.50 / 47.50
SD19: 47.30
54.00 / 65.50
SD20: 59.95
61.20 / 75.60
StKI-A: 62.80

## Slide 4
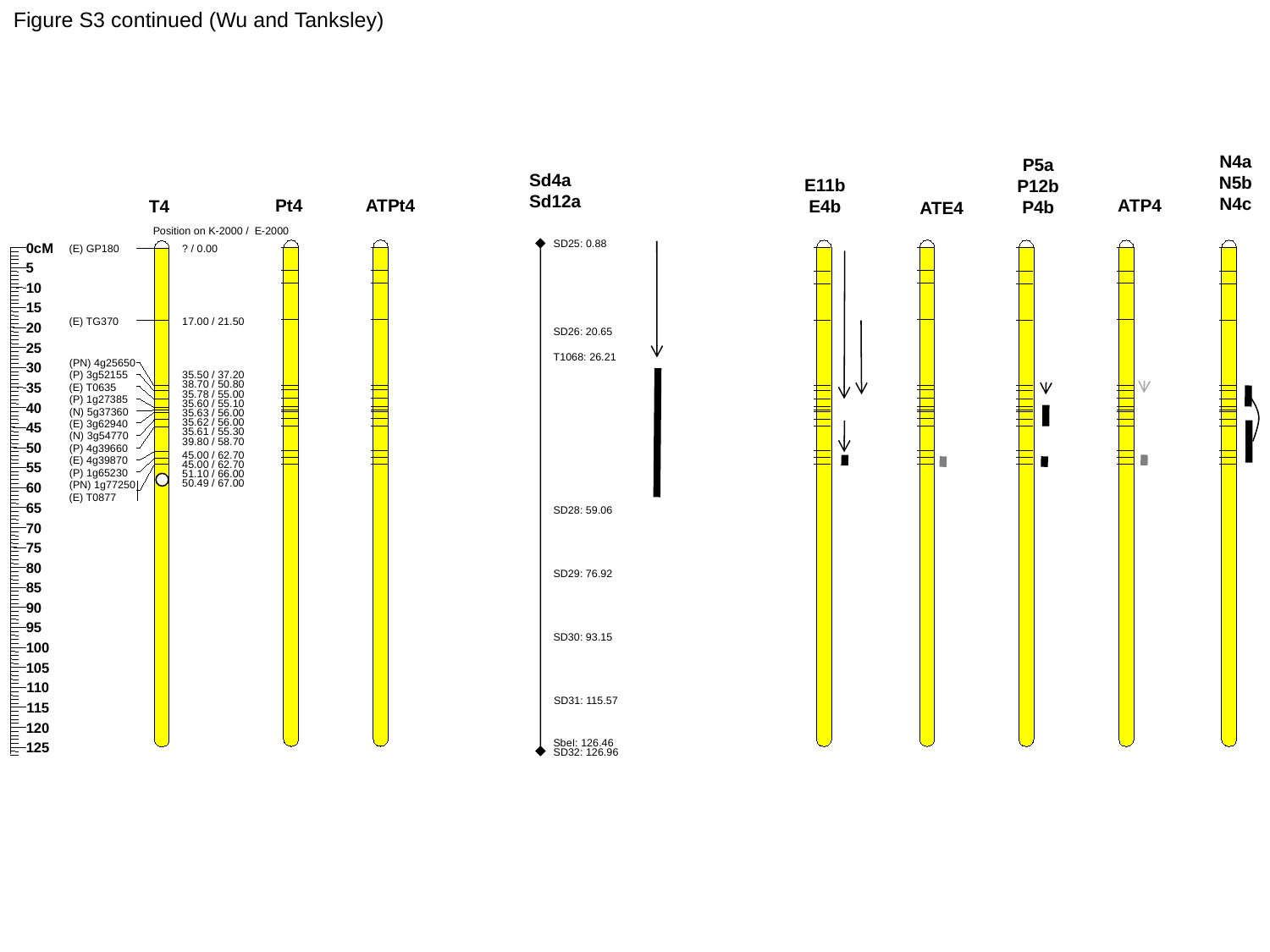

Figure S3 continued (Wu and Tanksley)
N4a
N5b
N4c
P5a
P12b
P4b
Sd4a
Sd12a
E11b
E4b
ATP4
Pt4
ATPt4
T4
(E) GP180
(E) TG370
(PN) 4g25650
(P) 3g52155
(E) T0635
(P) 1g27385
(N) 5g37360
(E) 3g62940
(N) 3g54770
(P) 4g39660
(E) 4g39870
(P) 1g65230
(PN) 1g77250
(E) T0877
ATE4
Position on K-2000 / E-2000
SD25: 0.88
? / 0.00
0cM
5
10
15
20
25
30
35
40
45
50
55
60
65
70
75
80
85
90
95
100
105
110
115
120
125
17.00 / 21.50
SD26: 20.65
T1068: 26.21
35.50 / 37.20
38.70 / 50.80
35.78 / 55.00
35.60 / 55.10
35.63 / 56.00
35.62 / 56.00
35.61 / 55.30
39.80 / 58.70
45.00 / 62.70
45.00 / 62.70
51.10 / 66.00
50.49 / 67.00
SD28: 59.06
SD29: 76.92
SD30: 93.15
SD31: 115.57
SbeI: 126.46
SD32: 126.96

## Slide 5
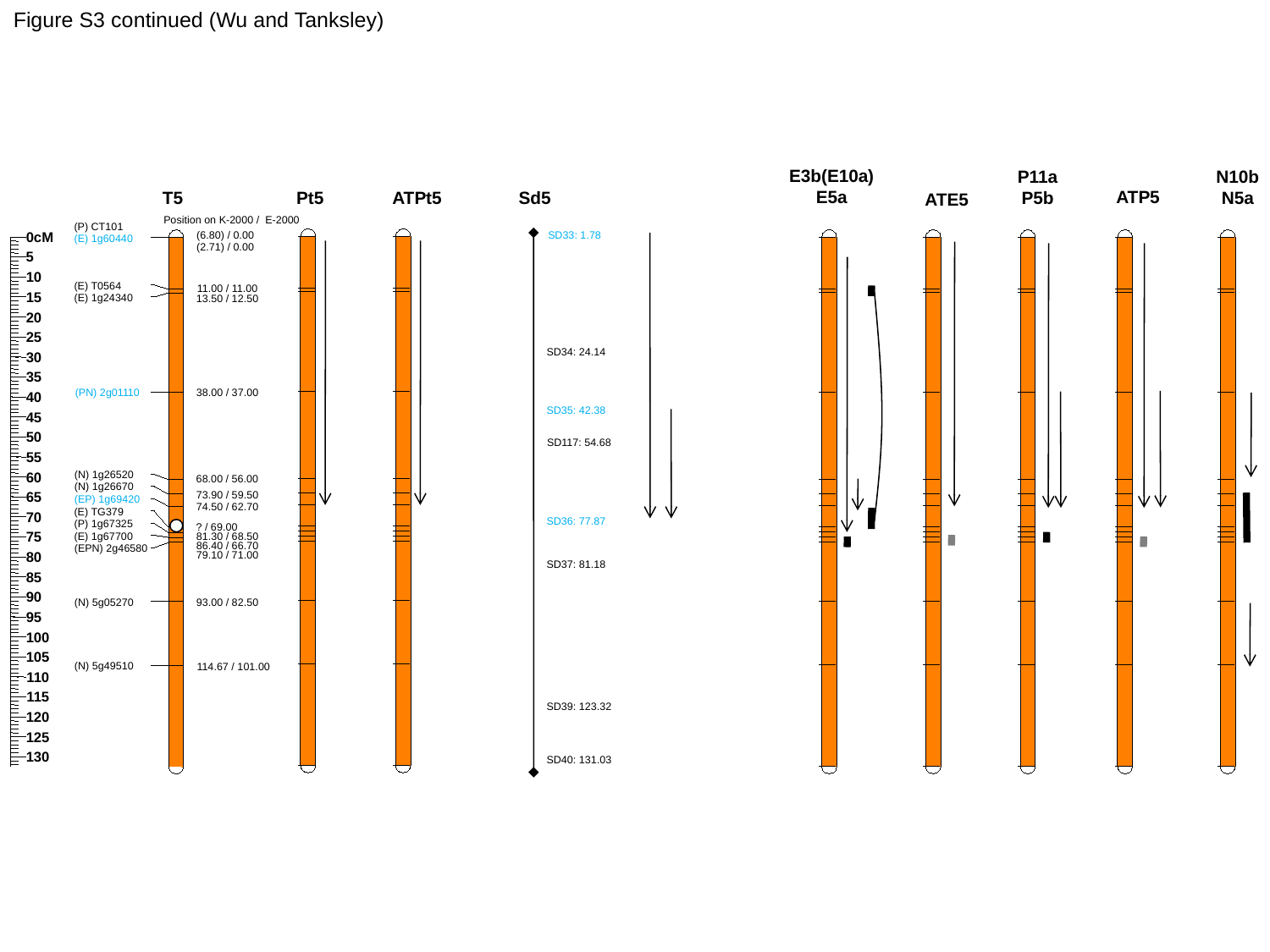

Figure S3 continued (Wu and Tanksley)
E3b(E10a)
E5a
P11a
P5b
N10b
N5a
ATP5
Sd5
T5
(P) CT101
(E) 1g60440
(E) T0564
(E) 1g24340
(PN) 2g01110
(N) 1g26520
(N) 1g26670
(EP) 1g69420
(E) TG379
(P) 1g67325
(E) 1g67700
(EPN) 2g46580
(N) 5g05270
(N) 5g49510
Pt5
ATPt5
ATE5
Position on K-2000 / E-2000
(6.80) / 0.00
SD33: 1.78
0cM
5
10
15
20
25
30
35
40
45
50
55
60
65
70
75
80
85
90
95
100
105
110
115
120
125
130
(2.71) / 0.00
11.00 / 11.00
13.50 / 12.50
SD34: 24.14
38.00 / 37.00
SD35: 42.38
SD117: 54.68
68.00 / 56.00
73.90 / 59.50
74.50 / 62.70
SD36: 77.87
? / 69.00
81.30 / 68.50
86.40 / 66.70
79.10 / 71.00
SD37: 81.18
93.00 / 82.50
114.67 / 101.00
SD39: 123.32
SD40: 131.03

## Slide 6
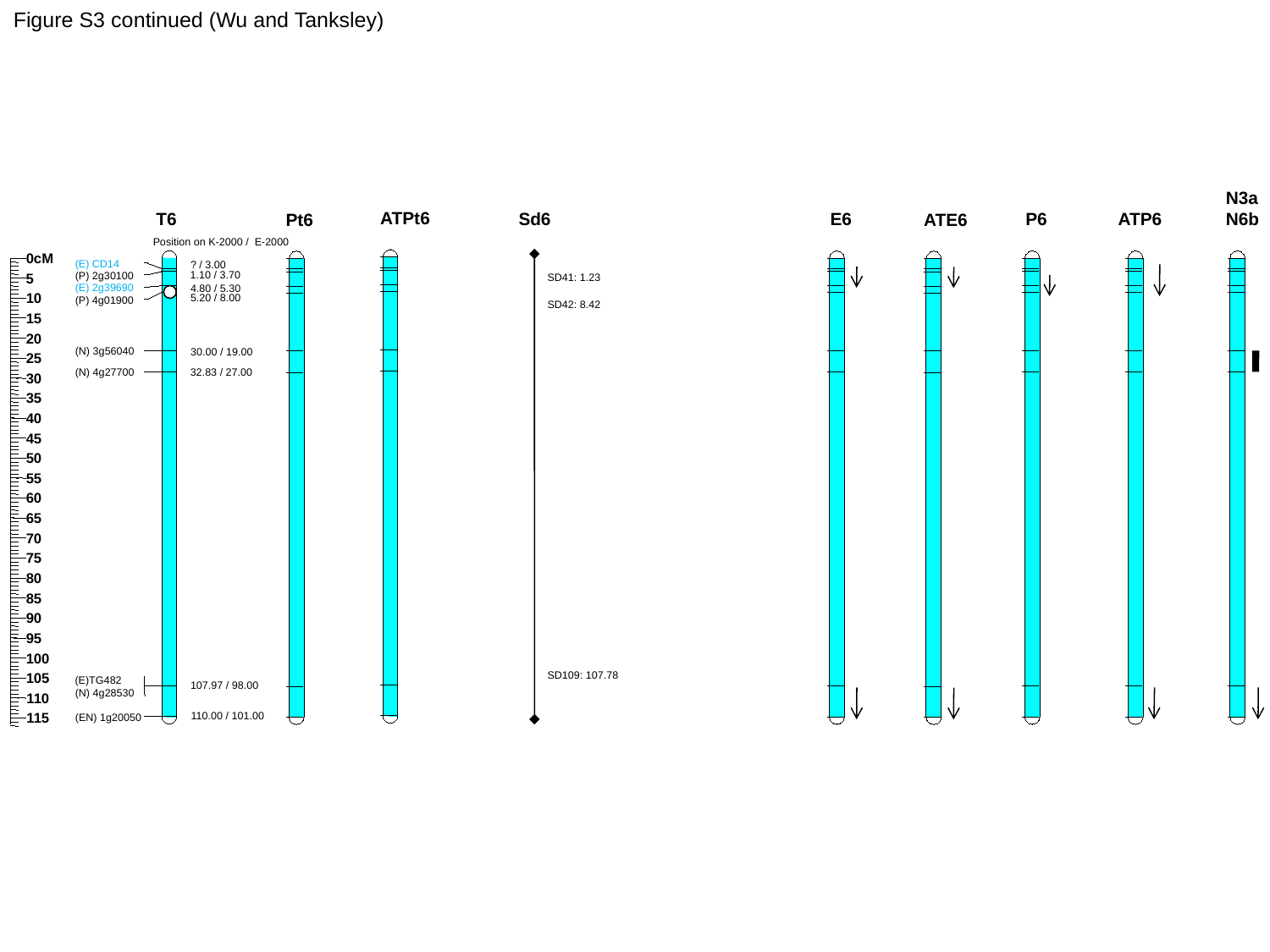

Figure S3 continued (Wu and Tanksley)
N3a
N6b
ATPt6
Sd6
T6
(E) CD14
(P) 2g30100
(E) 2g39690
(P) 4g01900
(N) 3g56040
(N) 4g27700
(E)TG482
(N) 4g28530
(EN) 1g20050
E6
P6
ATP6
Pt6
ATE6
Position on K-2000 / E-2000
0cM
5
10
15
20
25
30
35
40
45
50
55
60
65
70
75
80
85
90
95
100
105
110
115
? / 3.00
1.10 / 3.70
SD41: 1.23
4.80 / 5.30
5.20 / 8.00
SD42: 8.42
30.00 / 19.00
32.83 / 27.00
SD109: 107.78
107.97 / 98.00
110.00 / 101.00

## Slide 7
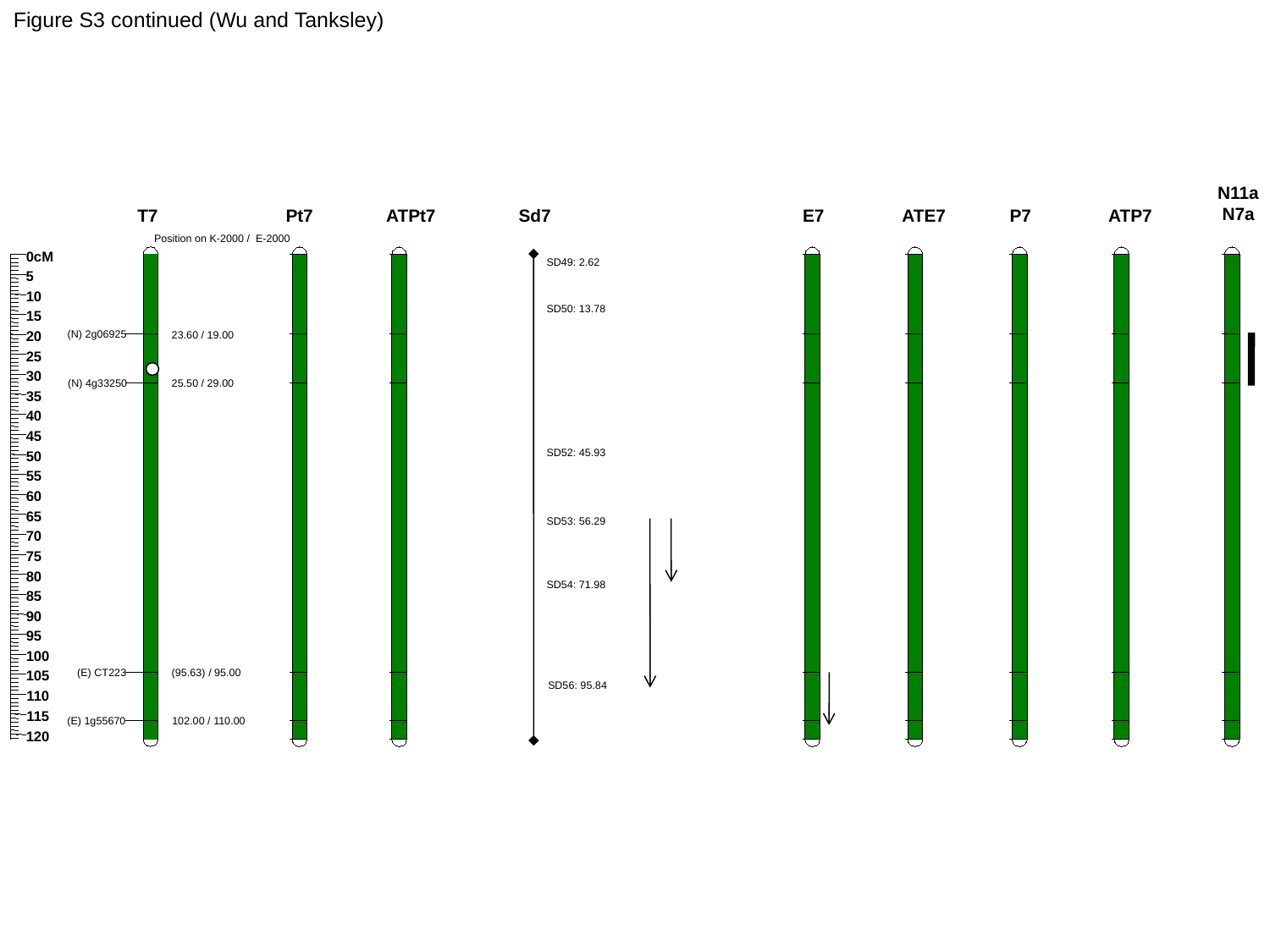

Figure S3 continued (Wu and Tanksley)
N11a
N7a
T7
(N) 2g06925
(N) 4g33250
(E) CT223
(E) 1g55670
Pt7
ATPt7
Sd7
E7
ATE7
P7
ATP7
Position on K-2000 / E-2000
0cM
5
10
15
20
25
30
35
40
45
50
55
60
65
70
75
80
85
90
95
100
105
110
115
120
SD49: 2.62
SD50: 13.78
23.60 / 19.00
25.50 / 29.00
SD52: 45.93
SD53: 56.29
SD54: 71.98
(95.63) / 95.00
SD56: 95.84
102.00 / 110.00

## Slide 8
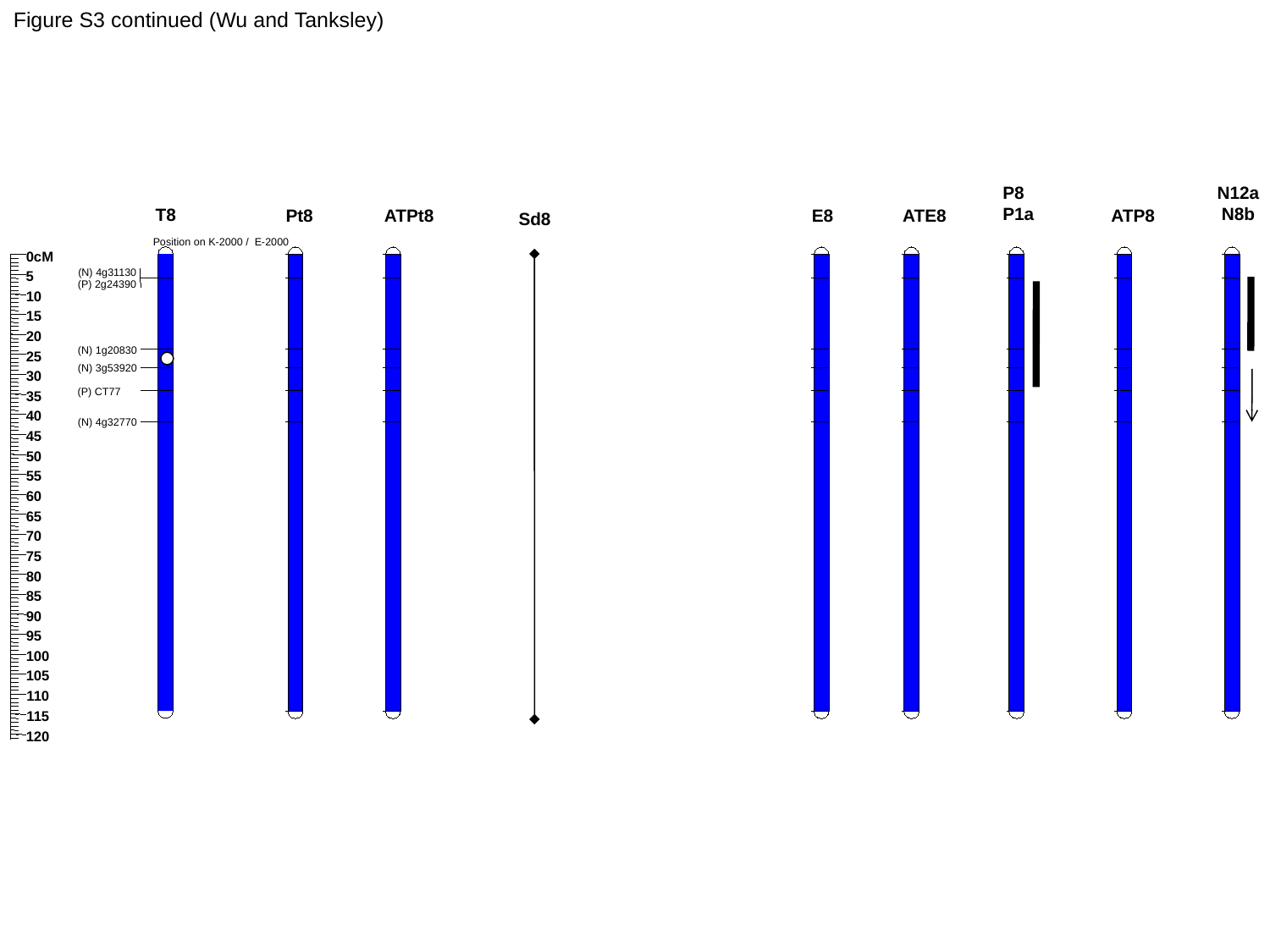

Figure S3 continued (Wu and Tanksley)
P8
P1a
N12a
N8b
T8
(N) 4g31130
(P) 2g24390
(N) 1g20830
(N) 3g53920
(P) CT77
(N) 4g32770
ATP8
Pt8
ATPt8
E8
ATE8
Sd8
Position on K-2000 / E-2000
0cM
5
10
15
20
25
30
35
40
45
50
55
60
65
70
75
80
85
90
95
100
105
110
115
120

## Slide 9
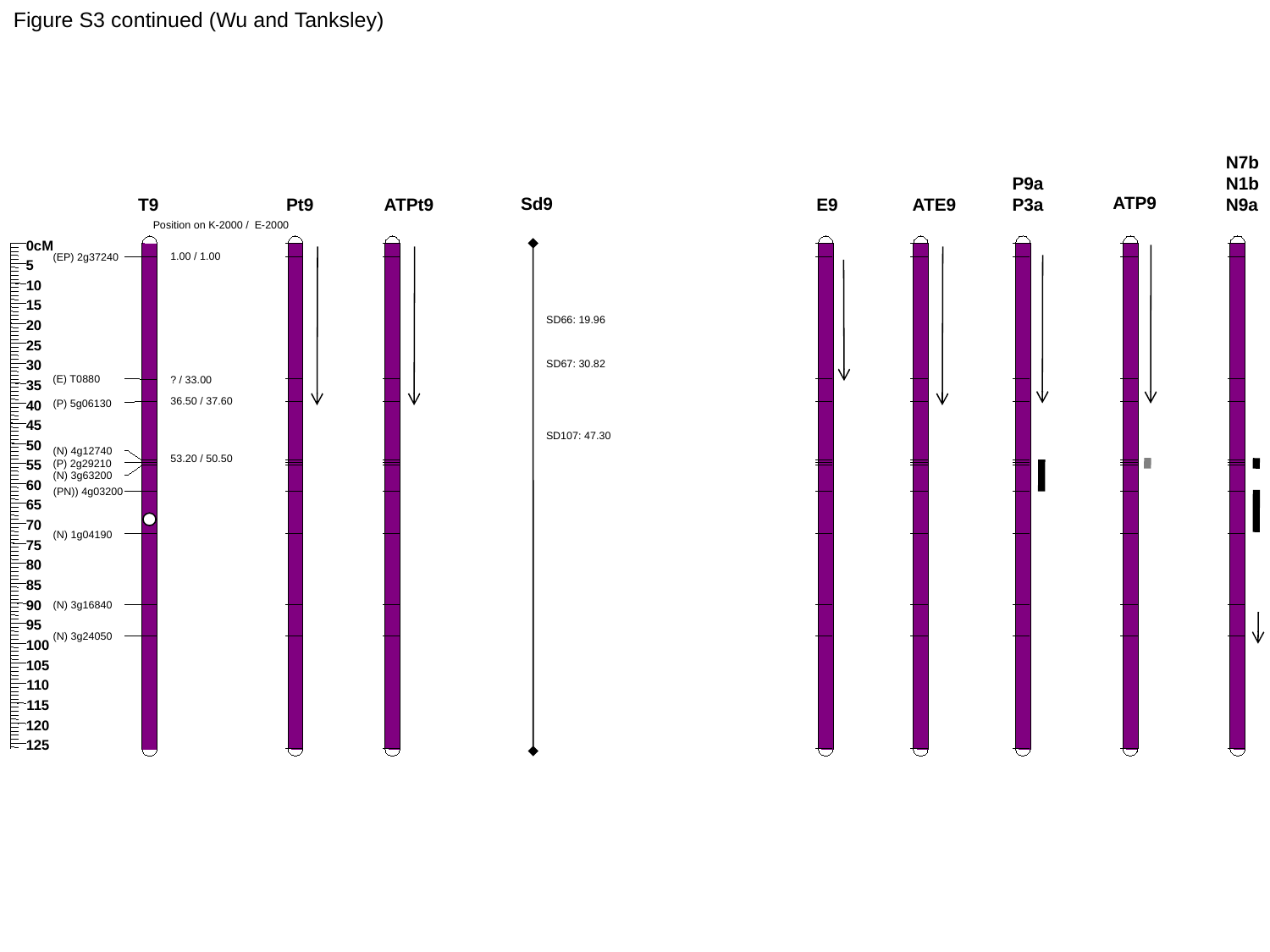

Figure S3 continued (Wu and Tanksley)
N7b
N1b
N9a
P9a
P3a
ATP9
Sd9
T9
(EP) 2g37240
(E) T0880
(P) 5g06130
(N) 4g12740
(P) 2g29210
(N) 3g63200
(PN)) 4g03200
(N) 1g04190
(N) 3g16840
(N) 3g24050
Pt9
ATPt9
E9
ATE9
Position on K-2000 / E-2000
0cM
5
10
15
20
25
30
35
40
45
50
55
60
65
70
75
80
85
90
95
100
105
110
115
120
125
1.00 / 1.00
SD66: 19.96
SD67: 30.82
? / 33.00
36.50 / 37.60
SD107: 47.30
53.20 / 50.50

## Slide 10
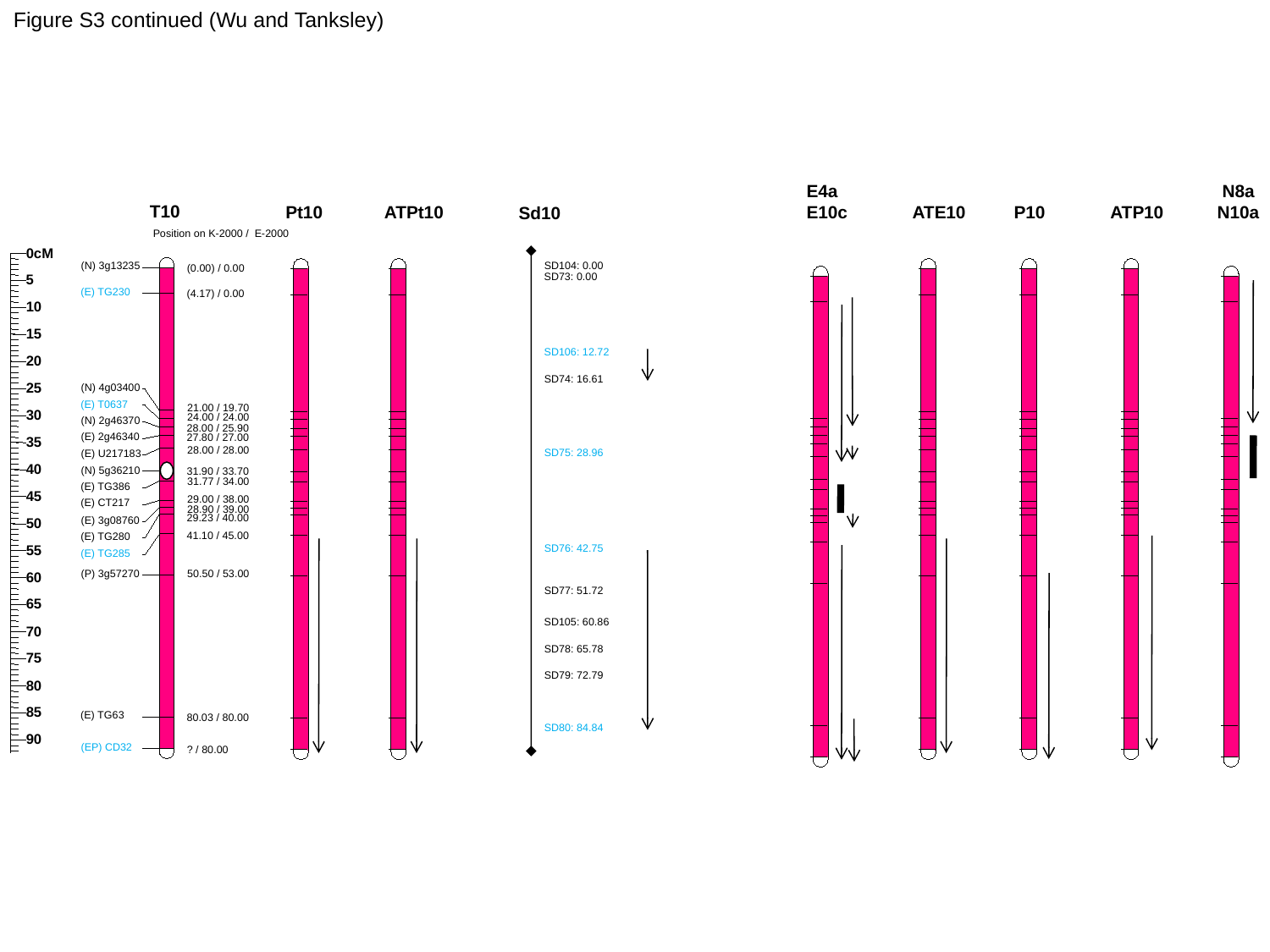

Figure S3 continued (Wu and Tanksley)
E4a
E10c
N8a
N10a
T10
(N) 3g13235
(E) TG230
(N) 4g03400
(E) T0637
(N) 2g46370
(E) 2g46340
(E) U217183
(N) 5g36210
(E) TG386
(E) CT217
(E) 3g08760
(E) TG280
(E) TG285
(P) 3g57270
(E) TG63
(EP) CD32
Pt10
ATPt10
ATE10
P10
ATP10
Sd10
Position on K-2000 / E-2000
0cM
5
10
15
20
25
30
35
40
45
50
55
60
65
70
75
80
85
90
SD104: 0.00
(0.00) / 0.00
SD73: 0.00
(4.17) / 0.00
SD106: 12.72
SD74: 16.61
21.00 / 19.70
24.00 / 24.00
28.00 / 25.90
27.80 / 27.00
28.00 / 28.00
SD75: 28.96
31.90 / 33.70
31.77 / 34.00
29.00 / 38.00
28.90 / 39.00
29.23 / 40.00
41.10 / 45.00
SD76: 42.75
50.50 / 53.00
SD77: 51.72
SD105: 60.86
SD78: 65.78
SD79: 72.79
80.03 / 80.00
SD80: 84.84
? / 80.00

## Slide 11
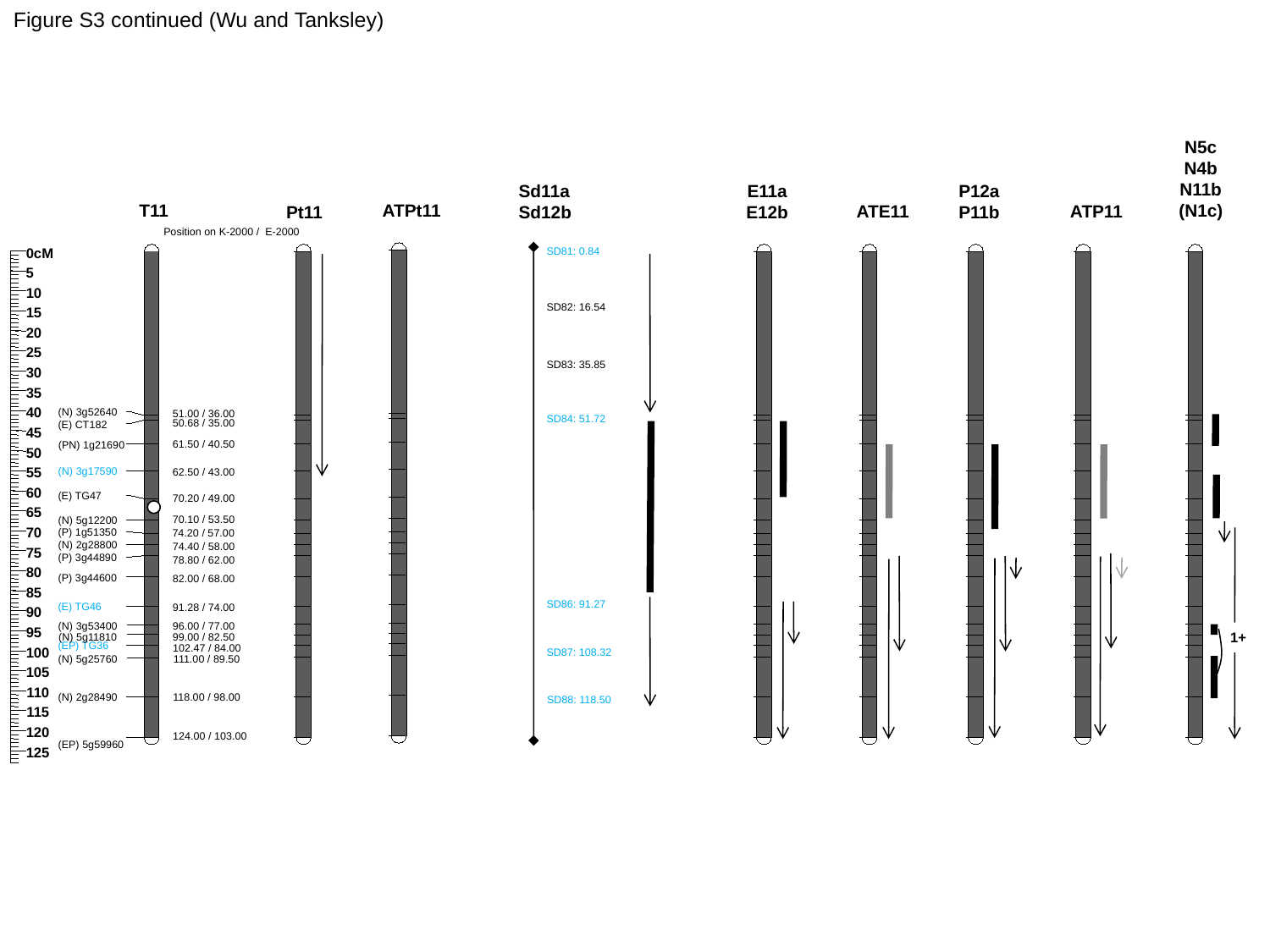

Figure S3 continued (Wu and Tanksley)
N5c
N4b
N11b
(N1c)
1+
Sd11a
Sd12b
E11a
E12b
P12a
P11b
T11
(N) 3g52640
(E) CT182
(PN) 1g21690
(N) 3g17590
(E) TG47
(N) 5g12200
(P) 1g51350
(N) 2g28800
(P) 3g44890
(P) 3g44600
(E) TG46
(N) 3g53400
(N) 5g11810
(EP) TG36
(N) 5g25760
(N) 2g28490
(EP) 5g59960
ATPt11
ATE11
ATP11
Pt11
Position on K-2000 / E-2000
SD81: 0.84
0cM
5
10
15
20
25
30
35
40
45
50
55
60
65
70
75
80
85
90
95
100
105
110
115
120
125
SD82: 16.54
SD83: 35.85
51.00 / 36.00
SD84: 51.72
50.68 / 35.00
61.50 / 40.50
62.50 / 43.00
70.20 / 49.00
70.10 / 53.50
74.20 / 57.00
74.40 / 58.00
78.80 / 62.00
82.00 / 68.00
SD86: 91.27
91.28 / 74.00
96.00 / 77.00
99.00 / 82.50
102.47 / 84.00
SD87: 108.32
111.00 / 89.50
118.00 / 98.00
SD88: 118.50
124.00 / 103.00

## Slide 12
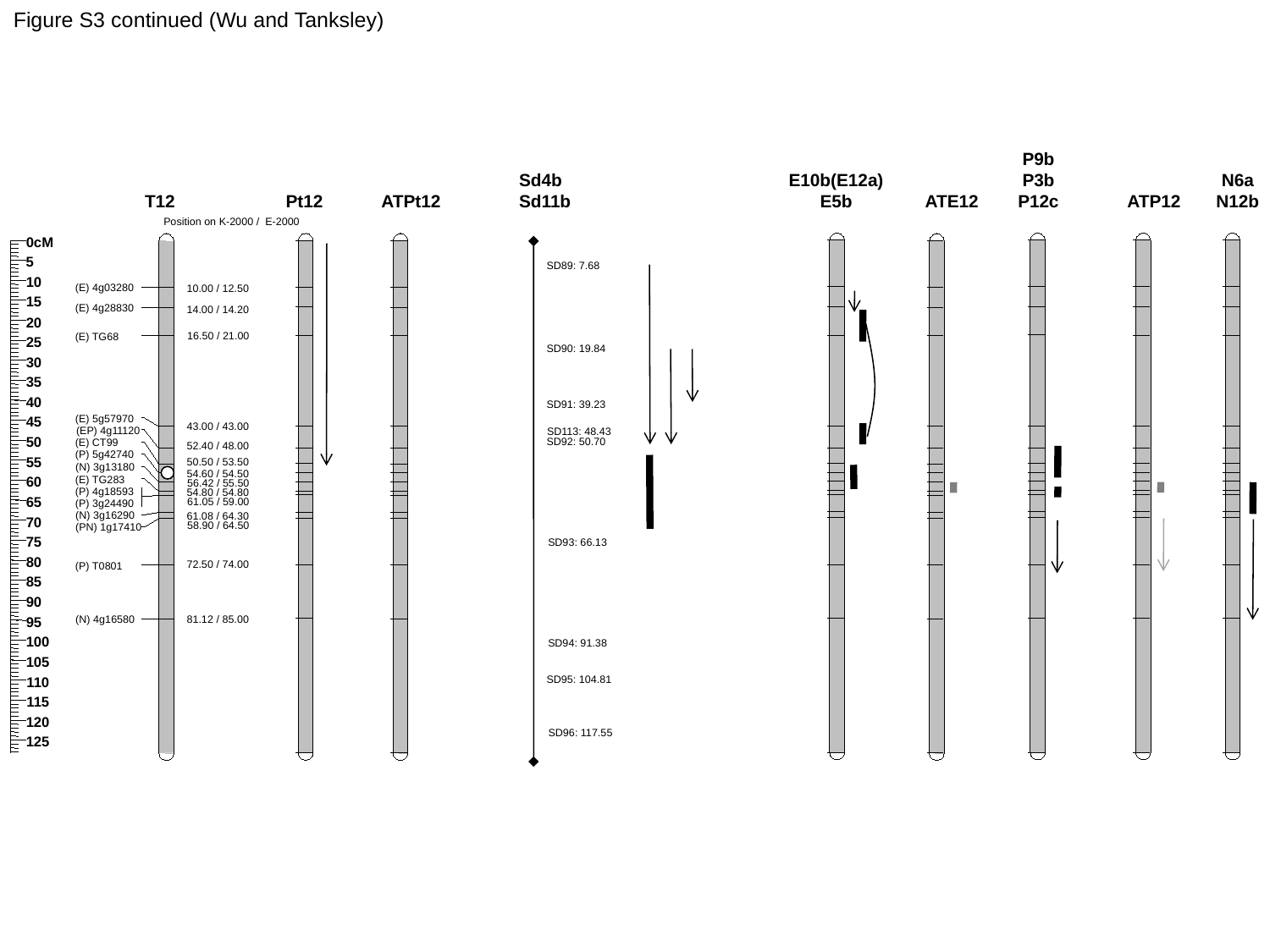

Figure S3 continued (Wu and Tanksley)
P9b
P3b
P12c
E10b(E12a)
E5b
Sd4b
Sd11b
N6a
N12b
T12
(E) 4g03280
(E) 4g28830
(E) TG68
(E) 5g57970
(EP) 4g11120
(E) CT99
(P) 5g42740
(N) 3g13180
(E) TG283
(P) 4g18593
(P) 3g24490
(N) 3g16290
(PN) 1g17410
(P) T0801
(N) 4g16580
Pt12
ATE12
ATP12
ATPt12
Position on K-2000 / E-2000
0cM
5
10
15
20
25
30
35
40
45
50
55
60
65
70
75
80
85
90
95
100
105
110
115
120
125
SD89: 7.68
10.00 / 12.50
14.00 / 14.20
16.50 / 21.00
SD90: 19.84
SD91: 39.23
43.00 / 43.00
SD113: 48.43
SD92: 50.70
52.40 / 48.00
50.50 / 53.50
54.60 / 54.50
56.42 / 55.50
54.80 / 54.80
61.05 / 59.00
61.08 / 64.30
58.90 / 64.50
SD93: 66.13
72.50 / 74.00
81.12 / 85.00
SD94: 91.38
SD95: 104.81
SD96: 117.55
